# Topography of the GLP-1/GLP-1 receptor system in the spinal cord of male mice

**DOI:** 10.1038/s41598-024-65442-1

**Published:** 2024-06-22

**Authors:** Yvette Ruska, Andrea Csibi, Beáta Dorogházi, Anett Szilvásy-Szabó, Petra Mohácsik, Zsuzsanna Környei, Ádám Dénes, Andrea Kádár, Zita Puskár, Erik Hrabovszky, Balázs Gereben, Gábor Wittmann, Csaba Fekete

**Affiliations:** 1 Laboratory of Integrative Neuroendocrinology, HUN-REN Institute of Experimental Medicine, Szigony Street 43, Budapest, 1083 Hungary; 2 Laboratory of Molecular Cell Metabolism, HUN-REN Institute of Experimental Medicine, Budapest, 1083 Hungary; 3 “Momentum” Laboratory of Neuroimmunology, HUN-REN Institute of Experimental Medicine, Budapest, 1083 Hungary; 4 Department of Anatomy, Histology and Embryology, Semmelweis University, Budapest, 1094 Hungary; 5 Laboratory of Reproductive Neurobiology, HUN-REN Institute of Experimental Medicine, Budapest, 1083 Hungary

**Keywords:** Obesity, Spinal cord, Obesity

## Abstract

Glucagon-like peptide-1 receptor (GLP-1R) agonists are now commonly used to treat type 2 diabetes and obesity. GLP-1R signaling in the spinal cord has been suggested to account for the mild tachycardia caused by GLP-1R agonists, and may also be involved in the therapeutic effects of these drugs. However, the neuroanatomy of the GLP-1/GLP-1R system in the spinal cord is still poorly understood. Here we applied in situ hybridization and immunohistochemistry to characterize this system, and its relation to cholinergic neurons. GLP-1R transcript and protein were expressed in neuronal cell bodies across the gray matter, in matching distribution patterns. GLP-1R-immunolabeling was also robust in dendrites and axons, especially in laminae II–III in the dorsal horn. Cerebrospinal fluid-contacting neurons expressed GLP-1R protein at exceedingly high levels. Only small subpopulations of cholinergic neurons expressed GLP-1R, including a subset of sympathetic preganglionic neurons at the rostral tip of the intermediolateral nucleus. GLP-1 axons innervated all regions where GLP-1R neurons were distributed, except laminae II–III. Scattered preproglucagon (*Gcg*) mRNA-expressing neurons were identified in the cervical and lumbar enlargements. The results will facilitate further studies on how GLP-1 regulates the sympathetic system and other autonomic and somatic functions via the spinal cord.

## Introduction

The glucagon-like peptide-1 (GLP-1)/GLP-1 receptor (GLP-1R) system has emerged in recent years as the main anti-diabetic and anti-obesity drug target^1,2^. GLP-1 is an incretin hormone released from enteroendocrine cells in response to eating that stimulates insulin secretion, slows gastric emptying and promotes satiety^1,3^. In addition, GLP-1 is also a neuropeptide produced by neurons in the lower brainstem^4–6^ that suppress eating in rodents^7–11^. The effects of GLP-1 is mediated by the GLP-1R that is widely expressed in several organs^12^, including the central nervous system (CNS)^6,13–16^. The weight-reducing effects of GLP-1R agonists are primarily attributed to their direct action on GLP-1R-expressing neurons in the hypothalamus and brainstem, although the exact mechanisms still remain to be investigated^2,12,17–19^.

While the GLP-1/GLP-1R system in the brain has been studied extensively, its anatomy and function in the spinal cord are poorly understood, despite the fact that the spinal cord has the highest concentration of GLP-1 within the CNS^9^. Pharmacogenetic ablation of GLP-1 neurons in the nucleus of the solitary tract (NTS) reduced the GLP-1 content of the spinal cord by almost 80%, indicating that brainstem neurons are the main, if not the only, source of GLP-1 in the spinal cord^9,20^. Axons originating from brainstem GLP-1 neurons make close contacts with sympathetic preganglionic neurons (SPN)^20^. This pathway has been suggested to increase heart rate under certain conditions (e.g., stress), as both activation of brainstem GLP-1 neurons and direct application of GLP-1 onto the spinal cord elicit tachycardia^21^. Based on these data, GLP-1R activation of SPNs has been proposed to be the mechanism underlying the mild tachycardia caused by GLP-1R agonist drugs^21^.

Currently only limited and conflicting data are available on the identity and distribution of cells expressing GLP-1R mRNA or protein in the spinal cord. RNA-Seq data from the mouse spinal cord indicate that the *Glp1r* transcript is expressed in several types of neurons^22,23^, and in situ hybridization in the rat labeled putative neurons across the gray matter^6^. In contrast, immunohistological studies, with little information on antibody specificity, report a homogenous pattern of GLP-1R staining in both the white and gray matter^24^, identifying GLP-1R-immunoreactive cells primarily as microglia^24–26^, but also as neurons^24–28^, oligodendrocytes^24^, and astrocytes^25^.

The goal of our present study was to elucidate the neuroanatomy of the GLP-1/GLP-1R system in the murine spinal cord to aid further functional studies of this system. We first examined the general distribution of *Glp1r* transcript with in situ hybridization, and the GLP-1R protein with immunohistological techniques, using a well-characterized, highly specific antibody^14,16,29^. Next, we correlated this distribution pattern with that of GLP-1 fibers, and investigated the existence of putative preproglucagon mRNA-expressing neurons within the spinal cord. Finally, we studied the presence of *Glp1r* mRNA or GLP-1R protein in cholinergic neurons, including SPNs.

## Results

### Distribution of Glp1r-expressing cells in the mouse spinal cord

**Figure 1:**
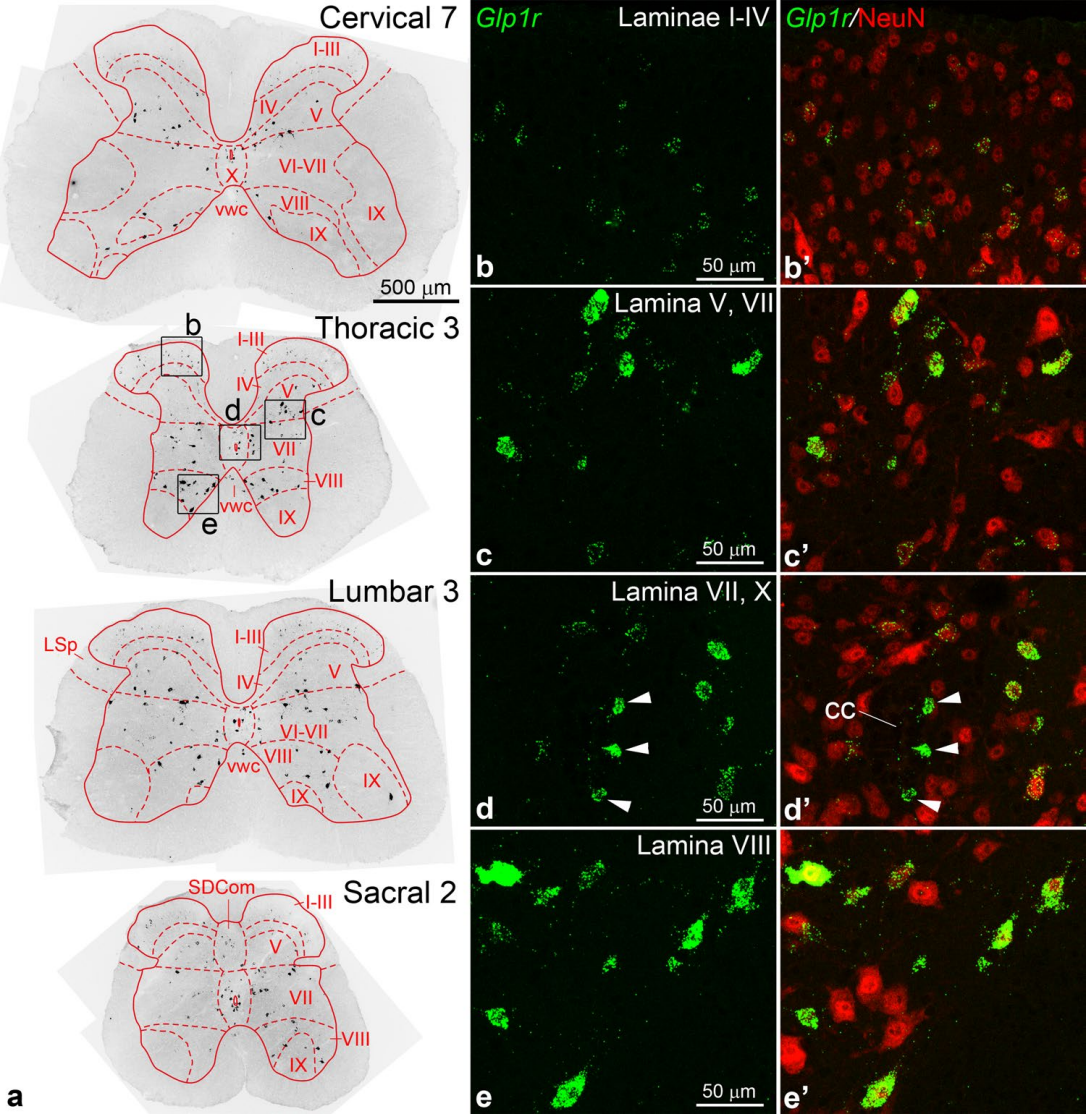
Distribution of *Glp1r* mRNA in the spinal cord. (**a**) Overview images show the distribution of *Glp1r*-expressing cells in whole spinal cord sections from cervical, thoracic, lumbar and sacral segments. For better visibility at this low magnification, the fluorescent signal of FISH was inverted, thus *Glp1r* labeling appears as black. Spinal cord laminae are indicated by Roman numerals. (**b**–**e’**) Higher magnification confocal images of the boxed areas from the thoracic segment in (**a**) show dual-labeling for *Glp1r* mRNA (green, FISH) and the neuronal marker NeuN (red, immunofluorescence). Most *Glp1r* cells contain NeuN, except those adjacent to the central canal (arrowheads), which are NeuN-negative. Note that *Glp1r* neurons in laminae I–IV express low levels of *Glp1r* mRNA. Abbreviations: cc, central canal; LSp, lateral spinal nucleus; SDCom, sacral dorsal commissural nucleus; vwc, ventral white commissure.

We studied the expression of *Glp1r* mRNA using fluorescent in situ hybridization (FISH). *Glp1r*-expressing cells were abundant at every level from the cervical to the coccygeal end of the spinal cord, predominantly in the gray matter, along with a few cells in the white matter (Fig. 1a). Most *Glp1r* positive cells had a clear neuronal morphology, and the hybridization signal often extended into their dendrites (Fig. 1c–e). Dual labeling with the neuronal marker NeuN confirmed the neuronal identity of the vast majority of *Glp1r*-expressing cells (Fig. 1b–e’), including those in the white matter. The only notable exception was a population of NeuN-negative *Glp1r* cells adjacent or near the central canal (Fig. 1d–d’).

The laminar distribution of *Glp1r*-expressing cells was similar from cervical to sacral segments, with few differences (Fig. 1a). A large population of *Glp1r* neurons with typically low hybridization signal were scattered across laminae I–IV of the dorsal horn (Fig. 1b–b’). The majority of these cells appeared to concentrate in the inner layer of lamina II and lamina III, but were rare in lamina I. Another major *Glp1r*-expressing neuron population was observed in lamina V, expressing moderate to high levels of *Glp1r* mRNA (Fig. 1c)*. Glp1r* neurons were numerous in the intermediate gray (lamina VII) and the central gray around the central canal (lamina X), displaying moderate to intense hybridization signal (Fig. 1d). In the ventral horn, *Glp1r* neurons typically had very intense hybridization signal, and were found primarily in lamina VIII (Fig. 1e). These neurons were more numerous in the thoracic than in cervical or lumbar segments, and conspicuous along the medial border of the rostral horn, and even in the ventral white commissure. Few *Glp1r* neurons were observed in lamina IX (Fig. 1a).

### Distribution of GLP-1R immunoreactivity

**Figure 2:**
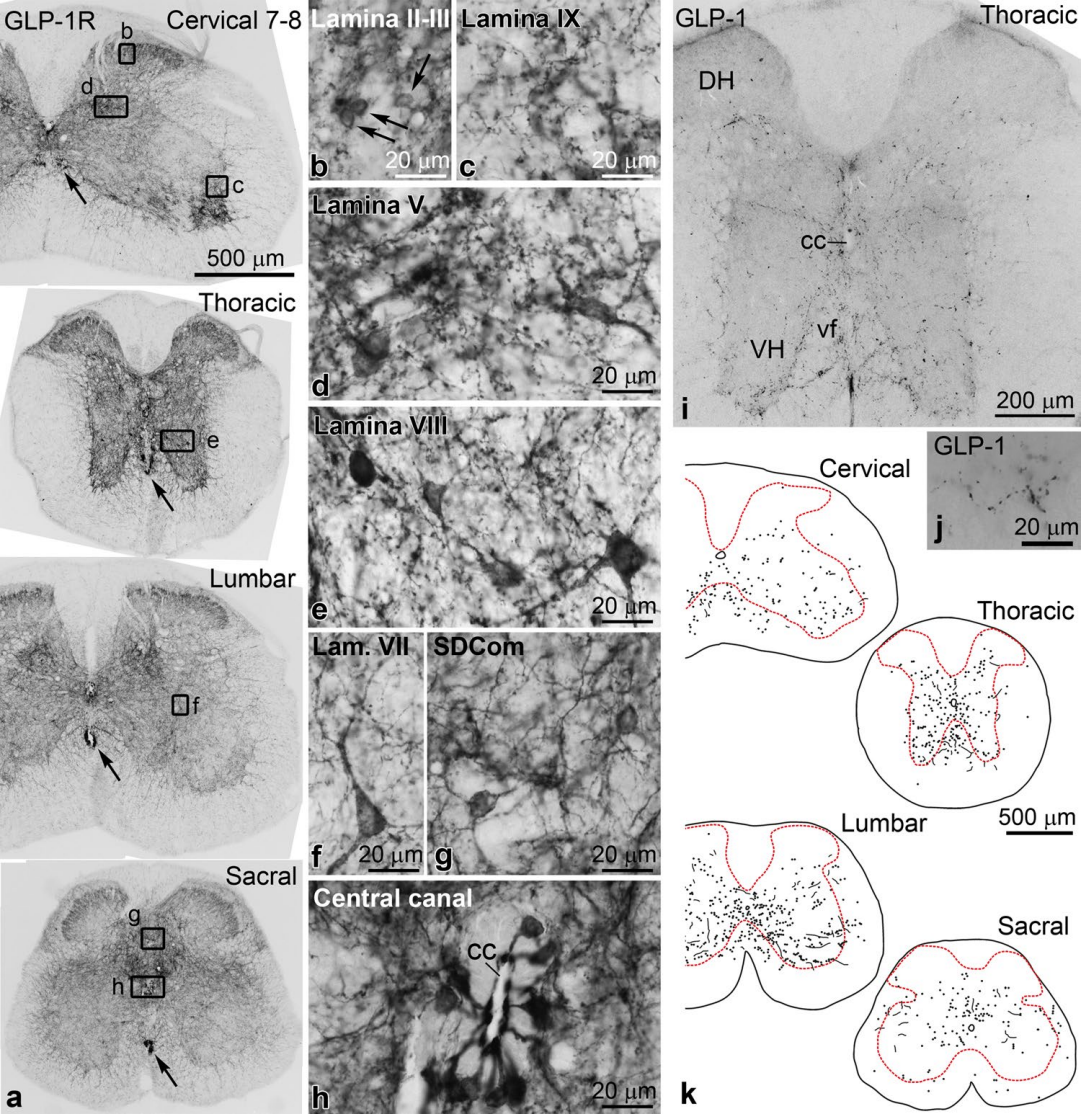
Distribution of GLP-1R- and GLP-1-immunoreactivity in the spinal cord. (**a**) Overview images of peroxidase immunohistochemistry (Ni-DAB) show the distribution of GLP-1R protein in cervical, thoracic, lumbar and sacral segments of the spinal cord. The arrows indicate the fiber bundles of CSF-contacting neurons in the ventral funiculus. High magnification images of the boxed areas are shown in (**b**–**h**). (**b**) GLP-1R labeling of small-sized perikarya (arrows) and processes in laminae II–III. (**c**) Beaded axons in lamina IX aggregate into dense patches in the tip of the ventral horn in the cervical enlargement. (**d**) GLP-1R perikarya in a dense network of varicose axons in the medial part of lamina V. (**e**) Intensely labeled perikarya and processes in lamina VIII of the thoracic segment. (**f**) GLP-1R labeling of a cell body and its long dendrites in lamina VII. (**g**) Perikarya and a network of dendrites in the sacral dorsal commissural nucleus. (**h**) Intense GLP-1R labeling of CSF-contacting neurons and their dendritic bulbs in the lumen of the central canal. (**i**) GLP-1 immunohistochemistry in a mid-thoracic segment of the spinal cord. Varicose GLP-1 axons provide moderate to dense innervation to the gray matter from the deep dorsal horn to the ventral horn, and the ventral funiculus. The more superficial layers of the dorsal horn (laminae I–III) receive only sparse GLP-1 fibers. (**j**) High magnification image of a varicose GLP-1 axon from the ventral horn. (**k**) Schematic drawings demonstrate the distribution density of GLP-1 fibers in cervical, thoracic, lumbar and sacral segments. Circles represent varicose GLP-1 axonal segments, lines represent some longer varicose segments. Abbreviations: cc, central canal; DH, dorsal horn; SDCom, sacral dorsal commissural nucleus; vf, ventral funiculus; VH, ventral horn.

To assess the general distribution of GLP-1R protein, we used chromogenic immunohistochemistry. Similar to the FISH results, GLP-1R-immunoreactive (IR) elements were observed primarily in the gray matter (Fig. 2a). Labeled elements included neuronal cell bodies and processes such as dendrites and beaded axons (Fig. 2b–h); neuronal processes accounted for most of the GLP-1R labeling. The distribution of GLP-1R-labeled perikarya closely matched that of *Glp1r* neurons detected by FISH. Small cell bodies were observed in laminae I–IV of the dorsal horn, particularly in a band spanning laminae II–III, embedded in a dense network of processes including varicose axons (Fig. 2b). Medium to large size GLP-1R perikarya were conspicuous from lamina V to VIII (Fig. 2d–f), and in the dorsal part of lamina X. Varicose GLP-1R axons could be observed in all these regions, but appeared particularly dense in laminae II–III and in the medial part of lamina V (Fig. 2d). Varicose GLP-1R axons formed large dense patches in the tip of the ventral horn in the cervical enlargement, in lamina IX (Fig. 2a,c). Dense GLP-1R immunostaining, mostly consisting of perikarya and dendrites, was observed in the sacral dorsal commissural nucleus (Fig. 2a,g).

The most intense GLP-1R labeling was observed in cells around the central canal, and in structures in the ventral funiculus, along the midline (Fig. 2a). The GLP-1R cells adjacent to the central canal extended a neurite into the lumen of the central canal (Fig. 2h), thus exhibiting the morphological features of cerebrospinal fluid (CSF)-contacting neurons^30,31^. The intensely labeled structures in the ventral funiculus correspond to the location of the fiber bundles of CSF-contacting neurons^31–33^.

### Subcellular localization of GLP-1R

**Figure 3:**
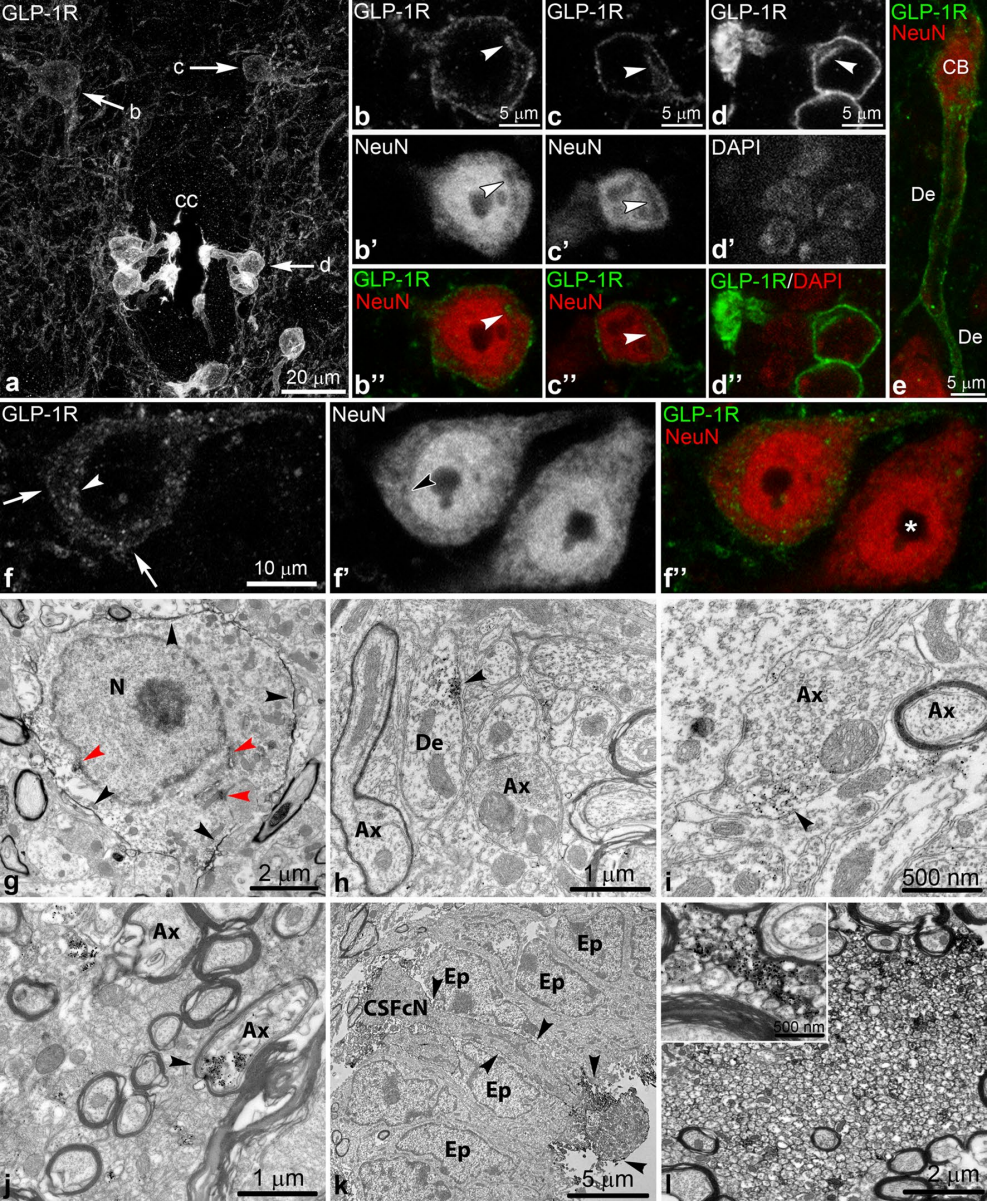
Subcellular distribution of GLP-1R. (**a**) GLP-1R immunofluorescence in lamina X of the thoracic spinal cord (Z-projection of optical sections, ~ 13 µm total thickness). Arrows indicate cells shown in high magnification in (**b**–**d’’**). (**b**–**d’’**) Single optical sections (0.7 µm thick) of two dorsal lamina X neurons (**b**, **c**) and a CSF-contacting neuron (**d**). GLP-1R is expressed on the cell membrane and concentrates intracellularly in large membrane compartments (arrowheads) that appear hollow in the NeuN staining that labels a soluble cytoplasmic/nuclear protein. For the CSF-contacting neuron, which is NeuN-negative, the DAPI-labeled nucleus is shown to demonstrate the perinuclear location of the membrane compartment. (**e**) GLP-1R immunolabeling on the dendrite of a lamina V neuron (0.7 µm thick optical section) (**f**–**f’’**) Single optical section of a GLP-1R neuron in lamina VIII that has partial GLP-1R labeling along the cell membrane (arrows) and a more diffuse intracellular labeling. The arrowhead points to GLP-1R labeling in the perinuclear membrane compartment. *indicates a GLP-1R negative neuron. (**g**–**l)** Immuno-electron microscopy for GLP-1R, using either the electron-dense Ni-DAB precipitate (**g**) or silver-intensified Ni-DAB (**h**–**l**). (**g**) GLP-1R-immunoreactivity is associated to the cell membrane (black arrowheads) and intracellular organelles (red arrowheads) in a neuronal perikaryon in the dorsal horn. (**h**–**j**) GLP-1R immunoreactivity in the dorsal horn also labeled dendrites (**h**), axon varicosities (**i**) and myelinated axons (**j**). (**k**) GLP-1R immunoreactivity is associated to the cell membrane (arrowheads) of a CSF-contacting neuron and particularly intense in the dendritic bulb. (**l**) Heavy GLP-1R immunoreactivity in a bundle of unmyelinated axons in the anterior funiculus, originating from CSF-contacting neurons. The inset shows the axon bundle at a higher magnification. Abbreviations: Ax, axon; CB, cell body; cc, central canal; CSFcN, CSF-contacting neuron; De, Dendrite; Ep, ependymal cell; N, neuronal nucleus.

We applied immunofluorescence and confocal microscopy as well as immuno-electron microscopy to examine the subcellular distribution of the GLP-1R. With confocal microscopy we observed that most labeled perikarya had pronounced fluorescent signal along the cell membrane that extended into the dendrites (Fig. 3a–e). In addition, it was common to observe intracellular labeling that often concentrated in a perinuclear band or in larger spots (Fig. 3b–d’’). These likely denote large membrane compartments such as the endoplasmic reticulum and/or Golgi compartment, as they appear hollow in the NeuN staining that labels the soluble cytoplasmic protein Rbfox3 (Fig. 3b–c’’)^34,35^. Some neurons had less membrane labeling, covering only portions of the cell surface, and the intracellular signal could appear more dispersed (Fig. 3f–f’’).

With immuno-electron microscopy in the dorsal horn, we observed GLP-1R-immunoreactivity associated to the cell membrane and intracellularly in neuronal perikarya (Fig. 3g). Membrane-associated or intracellular labeling was also observed in dendrites and axon varicosities (Fig. 3h,i), and in some myelinated axons (Fig. 3j). At the central canal, GLP-1R immunoreactivity was observed along the cell membrane of CSF-contacting neurons, with the heaviest labeling in the dendritic bulb in the central canal (Fig. 3k). The extremely intense GLP-1R immunoreaction in the anterior funiculus was localized in bundles of unmyelinated axons (Fig. 3l), confirming their identity as the axon bundles of CSF-contacting neurons^31–33^.

### GLP-1R immunoreactivity in glial cells

Since earlier publications reported GLP-1R immunoreactivity in glial cells^24–26^, we examined potential *Glp1r*/GLP-1R expression by astrocytes and microglia. At the ultrastructural level, GLP-1R immunoreactivity was observed intracellularly in a few fine processes that were reminiscent of glial processes (Supplementary Fig. S1). Using immunofluorescence, we could not detect GLP-1R in astrocyte cell bodies or main processes (Supplementary Fig. S1). GLP-1R-immunoreactivity was only observed in association with some thin astrocyte processes, but it could not be determined unequivocally whether GLP-1R-immunoreactivity was present on their surface or on a neighboring structure. *Glp1r* mRNA signal was not observed in astrocytes by FISH, although *Glp1r* was detected by RT-PCR from primary spinal cord astrocyte cultures isolated from P3 mice (Supplementary Fig. S1). We did not detect GLP-1R-immunoreactivity in microglia by immunohistochemistry or by flow cytometric analysis of isolated microglia (Supplementary Fig. S1).

### Distribution of GLP-1 fibers in the spinal cord

We correlated the distribution of GLP-1R-IR neuronal elements with the GLP-1 innervation of the spinal cord that originates primarily, if not exclusively, from lower brainstem neurons^20^. Using chromogenic immunohistochemistry to identify GLP-1 fibers, the distribution of GLP-1-IR axons in the spinal cord was essentially identical to the published distribution of yellow fluorescent protein (YFP)-containing axons in transgenic mice that express YFP under the preproglucagon (PPG or *Gcg*) promoter^20^. Briefly, the highest density of varicose GLP-1 axons was observed in the ventral horn and in the ventral funiculus, particularly in thoracic segments (Fig. 2i–k), largely overlapping with the distribution of GLP-1R neurons in lamina VIII. A large number of GLP-1 fibers was also present in lamina VII and lamina X (Fig. 2i,k). In the dorsal horn, lamina V received slightly less dense GLP-1 innervation. Modest density of GLP-1 axons was observed in lamina IV, and only scattered fibers were present in laminae I–III (Fig. 2i,k). In the sacral segment, the sacral dorsal commissural nucleus received moderate GLP-1 innervation (Fig. 2k).

### Gcg mRNA expression in the spinal cord

**Figure 4:**
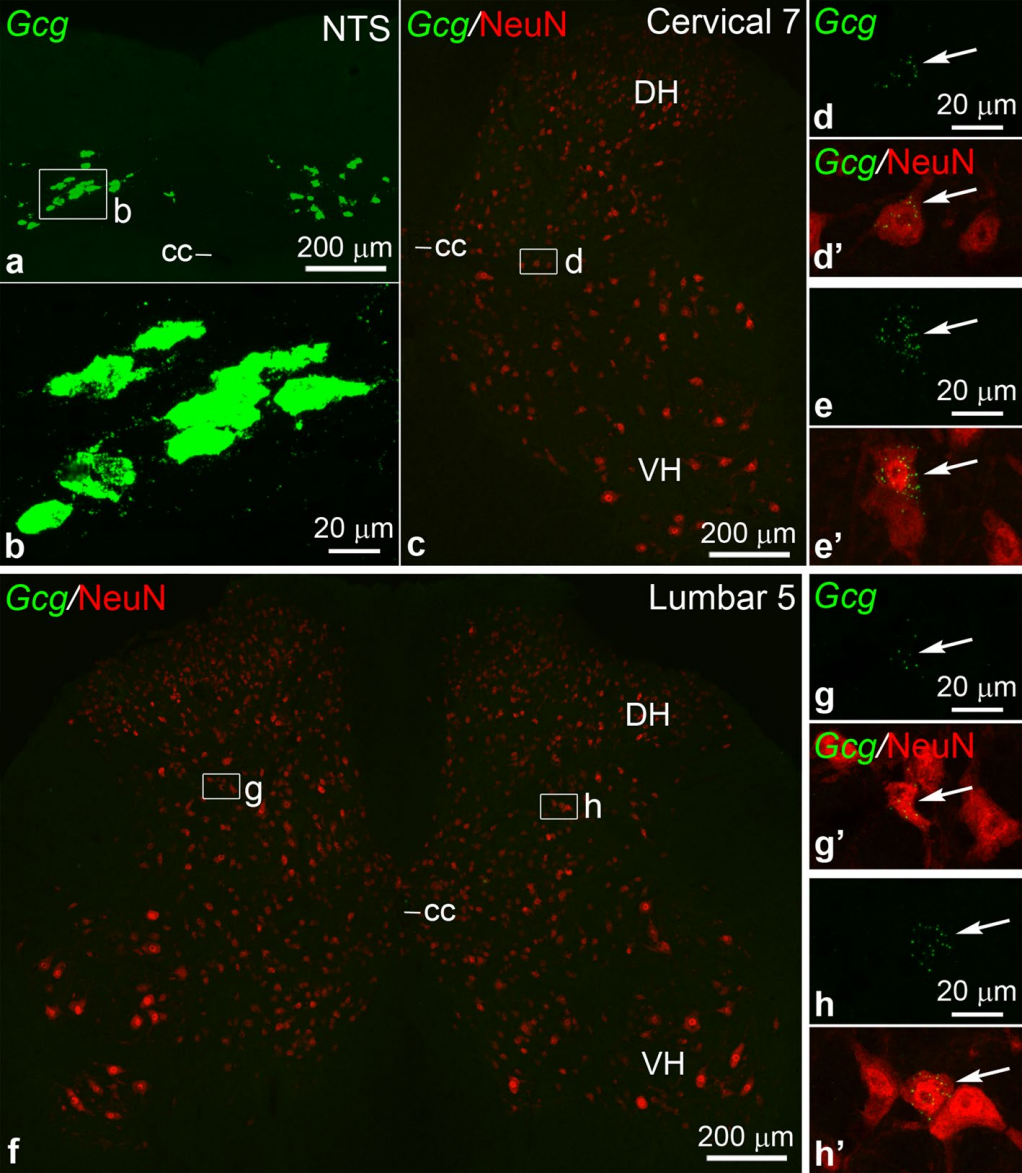
Low-level *Gcg* mRNA expression in spinal cord neurons. (**a**) FISH shows *Gcg* expression in the nucleus of the solitary tract in the caudal medulla. Boxed area is shown in higher magnification in (**b**). (**b**) High levels of *Gcg* mRNA is expressed in NTS neurons (Z-projection of optical sections, ~ 4 µm total thickness, for comparison to spinal cord *Gcg* neurons). (**c**) *Gcg* FISH (green) combined with NeuN immunofluorescence (red) in the cervical enlargement. The only *Gcg-*expressing cell is in the boxed area, shown in higher magnification in (**d**–**d’**). (**d**–**d’**) Low *Gcg* mRNA level is expressed in a NeuN positive neuron (Z-projection, ~ 4 µm total thickness) (**e**–**e’**) Another example of a *Gcg* neuron from the cervical enlargement (Z-projection, 6.6 µm total thickness) (**f**) *Gcg* FISH (green) and NeuN immunofluorescence (red) in the lumbar enlargement. The two *Gcg*-expressing cells found in the section are in the boxed areas, and shown in higher magnification in (**g**–**g’**) and **(h**–**h’**). (**g**–**h’**) Low *Gcg* mRNA levels in NeuN-positive neurons (arrows) (Z-projections, ~ 4 and 4.5 µm total thickness). Abbreviations: cc, central canal; DH, dorsal horn; NTS, nucleus of the solitary tract; VH, ventral horn.

Llewellyn-Smith and colleagues reported scattered YFP-expressing neurons in the deep dorsal horn of the lumbo-sacral spinal cord in PPG-YFP mice^20^. To investigate whether this finding represents genuine transcription of the *Gcg* gene, and thus possible GLP-1-producing neurons, or ectopic transgene expression, we performed FISH to detect *Gcg* mRNA. Scattered *Gcg* neurons (NeuN-positive) were observed in both the cervical and lumbar enlargements (Fig. 4c–h’). The *Gcg* expression of these neurons was very low, however, compared to *Gcg* mRNA levels in NTS neurons (Fig. 4a,b). In the cervical enlargement, *Gcg* neurons were found in 4 out of 8 mice (1–2 *Gcg* neurons between the C5–C7 segments, one section per segment examined), in lamina VI or VII (Fig. 4c–e’). In the lumbar enlargement, *Gcg* neurons were found in 5 out of 8 mice (1–4 *Gcg* neurons between the L3-S1 segments, one section per segment examined), in lamina V or VI (Fig. 4f–h’). In the eight cords examined we found only a single *Gcg* neuron in the thoracic segments (T12, lamina VII).

### Glp1r mRNA in cholinergic neurons

Axons of brainstem GLP-1 neurons were reported to form close appositions with cholinergic neurons in the autonomic nuclei that harbor SPNs: the intermediolateral nucleus (IML), intercalated nucleus (ICN) and the central autonomic area (CAA) (dorsal lamina X)^20^. To study *Glp1r* expression in cholinergic neurons, we applied dual FISH for *Glp1r* and the acetylcholine-synthesizing enzyme, choline acetyltransferase (*Chat*). Table 1 contains the cell counts of *Glp1r*^+^ *Chat*^+^ neurons in the different cell groups.

**Table 1:** Cell counts of *Glp1r*^+^ *Chat*^+^ neurons in different spinal cord regions.

Mouse ID#	Analyzed segments	IML C8 → T2	IML T12 → L1	ICN T1 → L2	CAA T1 → L2	CAA C1 → C8 L3 → Co	VH Lam VIII	SPSy	Other
7138	C3 → Co	8	1	0	7	0	2	0	1
7141	C5 → Co	16	1	0	7	0	2	1	0
7142	C4 → Co	10	2	2	10	1	1	0	2
7143	C6 → Co	10	0	3	7	0	1	1	2
7188	C1 → Co	13	1	11	9	4	3	1	1
7357	C3 → Co	11	2	4	17	4	3	0	1
7358	C3 → Co	8	0	2	11	0	1	0	2
7371	C6 → Co	5	3	1	9	2	2	0	0
Mean ± SEM		10.2 ± 1.9	1.3 ± 0.6	2.9 ± 2.1	9.6 ± 1.9	1.4 ± 1.0	1.9 ± 0.5	0.4 ± 0.3	1.0 ± 0.4

Cells were counted bilaterally, in one section per segment (for example, IML C8 → T2 was counted on three sections). C, cervical, CAA, central autonomic area; Co, coccygeal; ICN, intercalated nucleus; IML, intermediolateral nucleus; L, lumbar; SPSy, sacral parasympathetic nucleus; T, thoracic; VH Lam VIII, ventral horn lamina VIII. “Other” category includes *Glp1r*^+^ *Chat*^+^ neurons in lumbar lamina VII (4 cells), the cervical lateral funiculus (2 cells), cervical laminae II–III (1 cell) and lumbar and sacral somatic motor neurons in lamina IX (2 cells).

**Figure 5:**
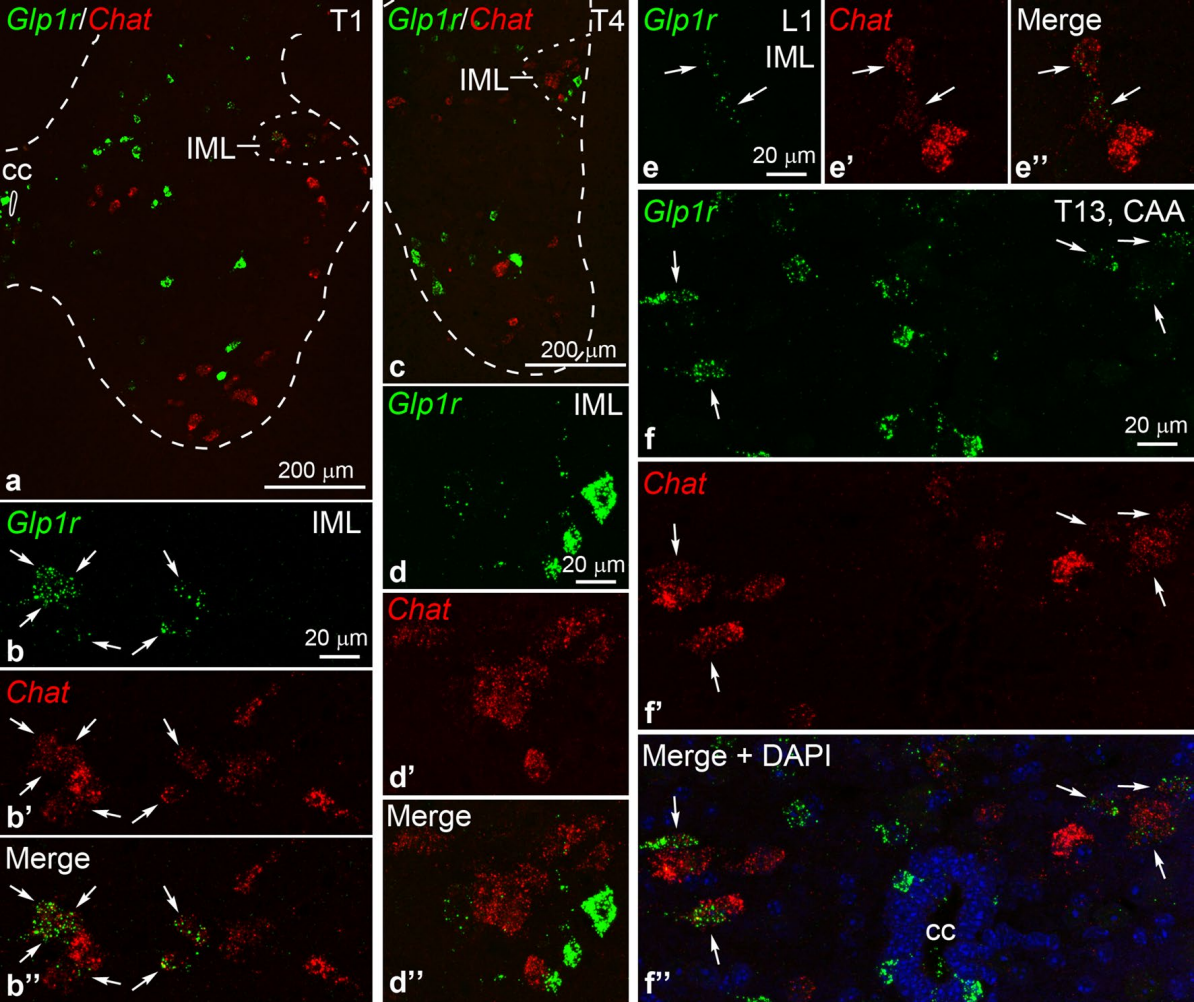
*Glp1r* expression in cholinergic neurons in autonomic nuclei of the spinal cord. (**a**–**b’’**) Dual FISH for *Glp1r* (green) and the cholinergic marker *Chat* (red) in the first thoracic segment (T1). The dashed line indicates the contours of the gray matter. (**b**–**b’’**) Higher magnification of the intermediolateral nucleus (IML) from (**a**) shows a subset of *Chat* neurons expressing *Glp1r* (arrows). (**c**–**d’’**) Images from the T4 segment show non-cholinergic neurons with high *Glp1r* mRNA levels in the IML in the vicinity of *Chat* neurons. (**d**–**d’’**) are high magnification confocal images of the IML in (**c**). (**e**–**e’’**) Some IML *Chat* neurons in the first lumbar segment (L1) express very low levels of *Glp1r* mRNA (arrows). (**f**–**f’’**) The images show dual-labeled *Glp1r*^+^ *Chat*^+^ neurons (arrows) among single-labeled *Chat*^+^ neurons in the central autonomic area (CAA), in the T13 segment. DAPI is shown in blue in **f’’**. The high magnification images (**b**–**b’’**, **d**–**f’’**) show Z-projections of confocal images (7–8 µm total thickness). Abbreviations: CAA, central autonomic area; cc, central canal; IML, intermediolateral nucleus.

Overall, *Glp1r*^+^ *Chat*^+^ neurons comprised a very small portion of any cholinergic population. In the IML, *Glp1r*^+^ *Chat*^+^ neurons were observed at its rostral and caudal end. The prominent population of these two was located rostrally, between the C8 and T2 segments (cervical 8 to thoracic 2), with the center in the T1 segment. These neurons expressed moderate levels of *Glp1r* mRNA and formed a subpopulation mainly in the medial and central portion of the IML (Fig. 5a–b’’): 38.9 ± 6.3% of *Chat*^+^ neurons in the T1, and 11.8 ± 4.0% in the T2 segment expressed *Glp1r* (n = 8 cords, 23.6 ± 4.2 *Chat*^+^ neurons in T1, 15.5 ± 2.0 in T2 counted in one section per segment). *Glp1r*^+^ *Chat*^+^ neurons were not observed in the pars funicularis of the C8-T2 IML. We did not observe *Glp1r*^+^ *Chat*^+^ neurons in the IML between the T3–T11 segments; however, neurons expressing high *Glp1r* mRNA level but not *Chat* were observed in the IML, mostly between T3–T7 (Fig. 5c–d’’). These cells probably represent interneurons in the IML^36^. A minor group of *Glp1r*^+^ *Chat*^+^ neurons was found in the caudal end of the IML, between the T12 and L1 segments. These neurons had very low *Glp1r* expression (Fig. 5e–e’’): only 1–3 were observed per cord, in 6 out of 8 mice (Table 1).

*Glp1r*^+^ *Chat*^+^ neurons in the ICN were few (Table 1), and rostro-caudally scattered. We observed a minor accumulation of dual-labeled neurons only in one cord, in the T4 segment (4 *Glp1r*^+^ *Chat*^+^ neurons per section). In the CAA, *Glp1r*^+^ *Chat*^+^ neurons were dispersed rostro-caudally between the T1 and L2 segments that contain both SPNs and cholinergic interneurons^36^. The overall number of these neurons was comparable to the number of *Glp1r*^+^ *Chat*^+^ neurons in the rostral IML group (Table 1). Minor accumulations of *Glp1r*^+^ *Chat*^+^ neurons (4–5 cells per section) in the CAA were observed in the T12–T13 (3 out of 8 mice) and T5 (2 out of 8 mice) segments (Fig. 5f–f’’). Sparse *Glp1r*^+^ *Chat*^+^ neurons were also present in cervical segments and below the L2 segment in the CAA; these cells are probably cholinergic interneurons^36^.

Very few *Glp1r*^+^ *Chat*^+^ neurons were found in other cholinergic cell populations. *Glp1r* transcript was extremely rare in somatic motor neurons in lamina IX (Table 1), although large *Glp1r* neurons were often found adjacent to large *Chat*^+^ motor neurons (Fig. 5a,c; Supplementary Fig. S2). We consistently observed some *Glp1r*^+^ *Chat*^+^ neurons in lamina VIII, mostly in thoracic segments, and almost always at the medial border of the ventral horn (Supplementary Fig. S2; Table 1). *Glp1r*^+^ *Chat*^+^ neurons were extremely rare in the sacral parasympathetic nucleus (Supplementary Fig. S2; Table 1).

### GLP-1R expression and GLP-1 innervation of cholinergic neurons

**Figure 6:**
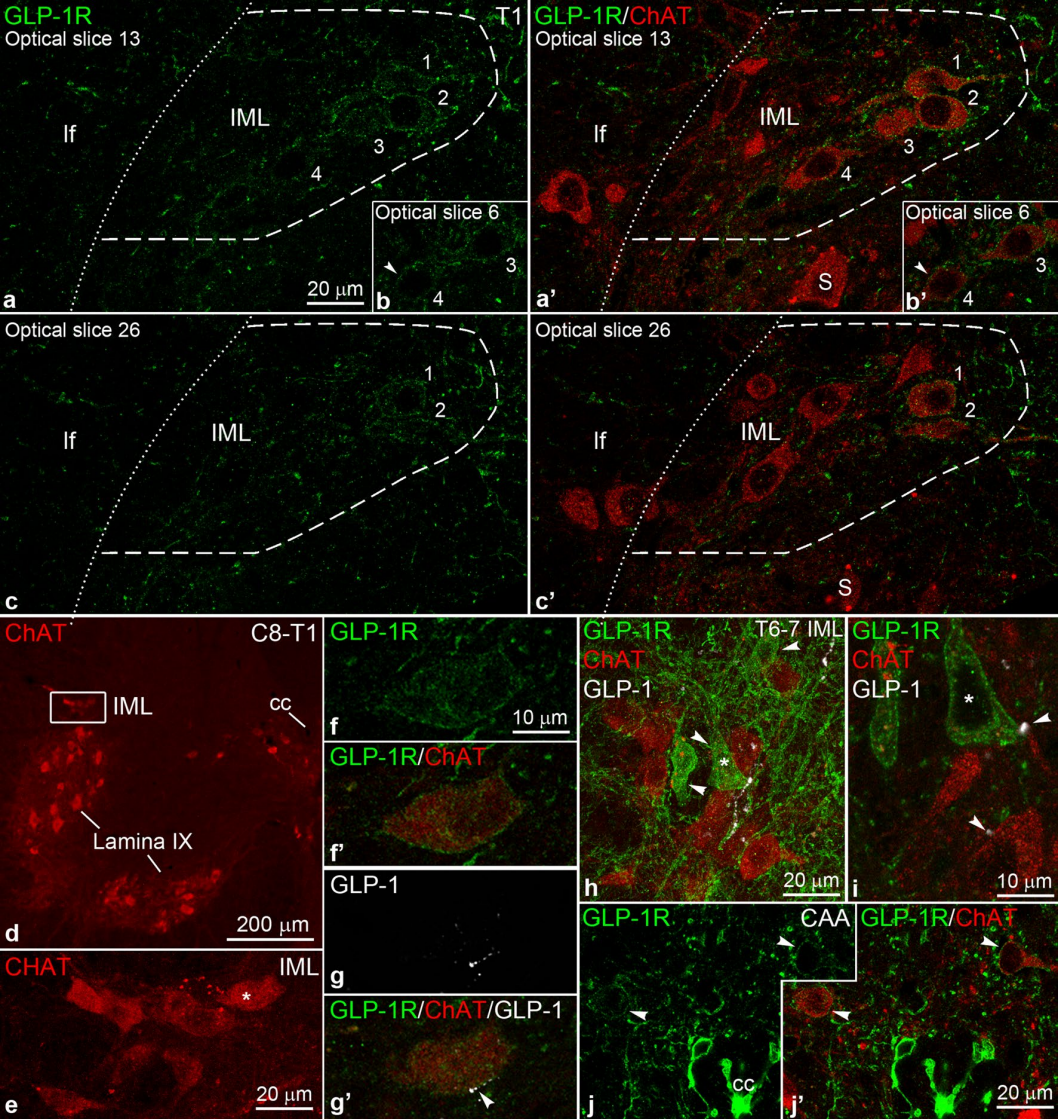
Subsets of cholinergic neurons in the IML and CAA express GLP-1R. (**a**–**c’**) Confocal images show dual immunofluorescence for GLP-1R (green) and ChAT (red) in the IML in the T1 segment. The single optical sections (0.82 µm thick) from three different Z-axis levels show four dual-labeled GLP-1R^+^ ChAT^+^ neurons (indicated by numbers 1–4), in the middle and central IML, and several GLP-1R-negative ChAT^+^ neurons. The arrowhead in (**b**–**b’**) points to GLP-1R labeling on the cell membrane of cell # 4. (**d**–**g’**) A GLP-1R^+^ ChAT^+^ neuron in the C8-T1 IML receives close appositions from a GLP-1 fiber (white in **g**–**g’**). (**f**–**f’**) shows a single optical section where GLP-1R label is pronounced on the cell membrane; (**g**–**g’**) shows a different optical section where the GLP-1 fiber is adjacent to the cell body. The location of this cell is shown in (**d**) (boxed area), and (**e**) (*indicates the cell). (**h**–**i**) Triple immunofluorescence for GLP-1R (green), ChAT (red) and GLP-1 (white) in the mid-thoracic IML. Z-projection of confocal images (~ 18 µm total thickness) in (**h**) shows three non-cholinergic neurons in the IML with intense GLP-1R labeling (arrowheads). A single optical section in (**i**) shows that GLP-1 varicosities (white) form close appositions (arrowheads) with the *-labeled GLP-1R neuron, and a ChAT neuron. (**j**–**j’**) A single confocal optical section (0.82 µm thick) shows two GLP-1R^+^ ChAT^+^ neurons (arrowheads) in the CAA from a mid-thoracic segment. Abbreviations: cc, central canal; lf, lateral funiculus; IML, intermediolateral nucleus; S, somatic motor neuron.

To confirm the presence of GLP-1R protein in cholinergic neurons and study their relationship to GLP-1 axons, we performed triple immunofluorescence for ChAT, GLP-1R and GLP-1. In agreement with the FISH results, we observed GLP-1R^+^ ChAT^+^ neurons in the IML in the C8-T2 segments (Fig. 6a–c’). These neurons had moderate GLP-1R immunolabeling on the cell membrane and lighter intracellular labeling (Fig. 6a–c’). In the T1 segment, 25.4 ± 2.5% of IML ChAT neurons were immunolabeled for GLP-1R (n = 3 cords, 113.3 ± 12.0 CHAT neurons counted in 4 sections from each cord). GLP-1R^+^ ChAT^+^ neurons were more frequent in the medial and central part of the IML, less common laterally, and almost never observed in the funicular part (Fig. 6a–c’). In some cases, we could trace long dendritic segments of GLP-1R^+^ ChAT^+^ neurons that extended medially toward the central gray matter. The GLP-1 innervation of the IML in the C8-T2 segments was relatively sparse, but we observed some GLP-1R^+^ ChAT^+^ neurons that received close appositions from GLP-1 axons (10 out of 123 GLP-1R^+^ ChAT^+^ neurons) (Fig. 6d–g’).

We did not observe GLP-1R^+^ ChAT^+^ neurons in the IML in any other segments (Fig. 6h; Supplementary Fig. S3), including the T12-L1 segments where few *Glp1r*^+^ *Chat*^+^ neurons were detected by FISH. The mid-thoracic IML appeared to receive more GLP-1 fibers than the T1 IML, and GLP-1 fibers were frequently apposed to the cell body or dendrites of these GLP-1R-negative ChAT neurons (Fig. 6h–i), confirming the results of Llewellyn-Smith and colleagues^20^. In accordance with the FISH results, we observed some very intensely labeled GLP-1R^+^ but ChAT-negative neurons in the IML in the upper- and mid-thoracic segments (Fig. 6h). Some of these neurons received appositions from GLP-1 varicosities (Fig. 6i). Of note, ChAT neurons at all segments were frequently contacted by GLP-1R axon varicosities (Supplementary Fig. S3), raising the possibility that GLP-1 may affect SPNs indirectly via these presynaptic terminals.

In the ICN and CAA, we observed GLP-1R^+^ ChAT^+^ neurons with a frequency similar to what the dual FISH results suggested (Fig. 6j–j’). GLP-1 axons were apposed to the vast majority, 84.4 ± 1.4%, of these neurons (17.7 ± 3.4 GLP-1R^+^ ChAT^+^ neurons examined in n = 3 cords, 37 CAA and 16 ICN neurons in total), often establishing multiple varicosities on their dendrites or cell body (Supplementary Fig. S4). We also confirmed that ChAT^+^ neurons in the sacral parasympathetic nucleus lack GLP-1R (n = 3 cords, 2 sections examined from each) (Supplementary Fig. S5).

### CSF-contacting neurons express Glp1r and receive close appositions from GLP-1 axons

**Figure 7:**
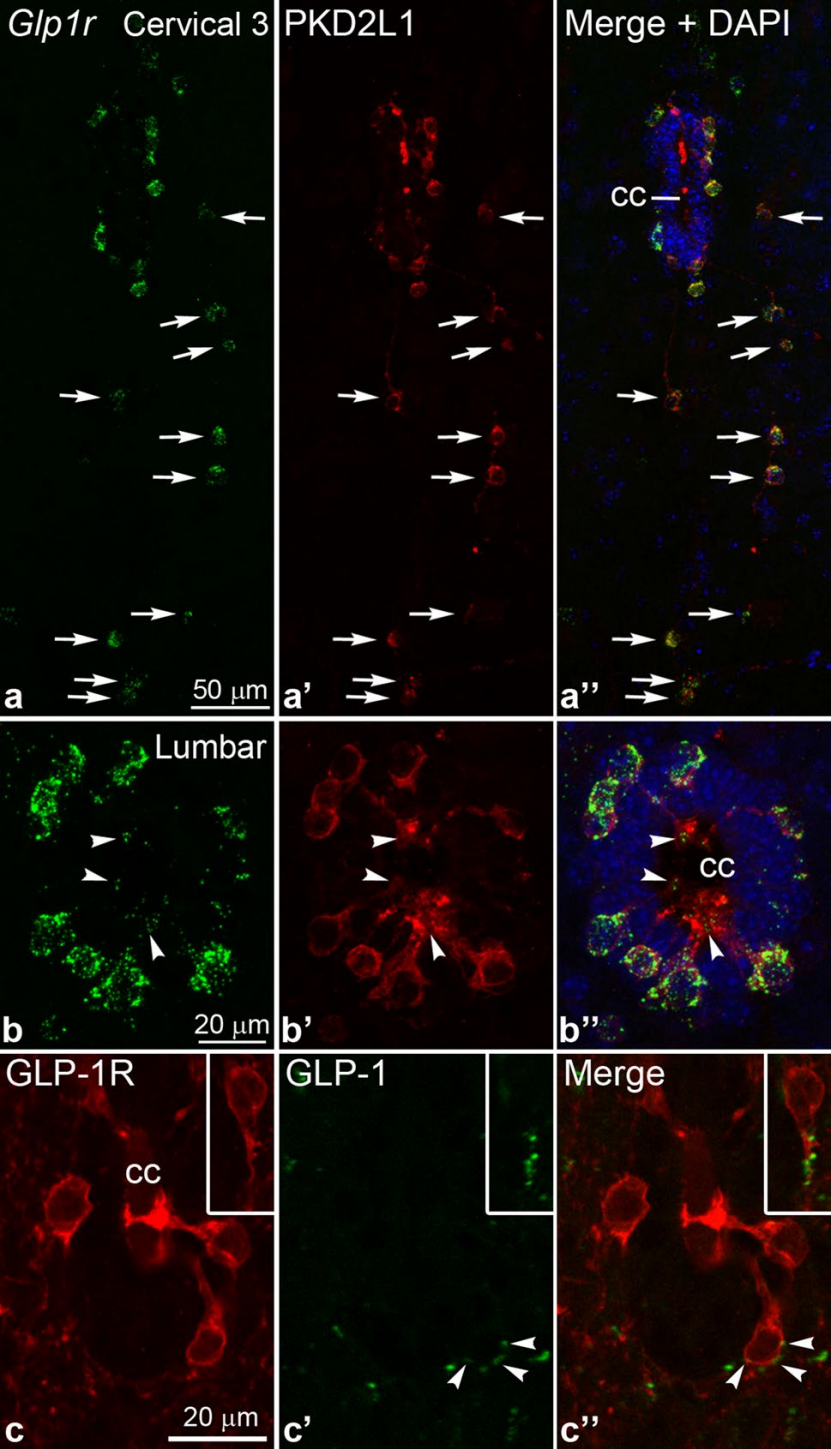
CSF-contacting neurons express *Glp1r* and receive close appositions from GLP-1 axons. (**a**–**b’’**) *Glp1r* FISH (green) combined with immunofluorescence for PKD2L1 (red), the marker for CSF-contacting neurons. (**a**–**a’’**) The confocal image (Z-projection) from the third cervical segment shows that all PKD2L1 positive neurons express *Glp1r* mRNA, both the ones adjacent to central canal, and those distant from it (arrows). (**b**–**b’’**) The confocal image (Z-projection) from the lumbar segment shows that all PKD2L1 neurons express *Glp1r*. *Glp1r* mRNA is also detected in their dendritic protrusions in the central canal (arrowheads). DAPI is shown in blue. (**c**–**c’’**) Dual immunofluorescence for GLP-1R (red) and GLP-1 (green) demonstrates that GLP-1 axon varicosities are closely apposed to the cell body of a CSF-contacting neuron (arrowheads). The inset shows a CSF-contacting neuron with GLP-1 varicosities apposed to its main dendrite. Single optical sections are shown. Abbreviations: cc, central canal.

Since CSF-contacting neurons were labeled with GLP-1R immunoreactivity at all spinal cord levels, we intended to confirm whether all CSF-contacting neurons express GLP-1R. However, dual immunofluorescence for GLP-1R and the marker for CSF-contacting neurons, PKD2L1 ^33^, was not feasible, because both antibodies are detected with anti-rabbit secondary IgGs (the GLP-1R antibody is a “rabbitized” mouse antibody). Therefore, we performed FISH for *Glp1r* and co-labeled the sections with the PKD2L1 antibody. All observed PKD2L1 cells expressed *Glp1r* (150 ± 10 PKD2L1 cells counted in 3 cords, examined from C3 to the coccygeal end), including PKD2L1 neurons located at a distance from the central canal^37,38^ (Fig. 7a–b’’). We also confirmed that CSF-contacting neurons are the NeuN-negative or weak NeuN positive *Glp1r* cells that we observed in the initial FISH experiment (Supplementary Fig. S6). Of note, we detected *Glp1r* mRNA also in the dendritic protrusions of CSF-contacting neurons (Fig. 7b–b’’), which often displayed the most intense GLP-1R immunolabeling (Figs. 2h, 3a).

We also studied whether GLP-1 fibers form close appositions with CSF-contacting neurons. Examined on confocal Z-stack images from the mid-thoracic segments, 36.1 ± 5.0% of CSF-contacting neurons received at least one close apposition from a GLP-1 axon on their cell body or proximal dendrite (n = 3 cords, 53 ± 5.7 CSF-contacting neurons examined on 6 sections from each cord) (Fig. 7c–c’’). This might be an underestimate of the real number, however, as many of the examined cell bodies were not contained entirely in the 25 µm-thick sections.

## Discussion

### Validity of the findings

The widespread distribution of *Glp1r* mRNA-containing neurons in the spinal cord is in agreement with published RNA-Seq data demonstrating *Glp1r* expression in multiple neuronal types of the spinal cord^22,23^. We observed a similar distribution pattern of *Glp1r mRNA-*containing cells compared to the earlier data from the rat^6^. However, we also identified a major population of *Glp1r* neurons in laminae II–III of the dorsal horn that was not detected earlier. We believe that this difference is due to the sensitivity of detection as these cells express very low levels of *Glp1r* mRNA.

To detect GLP-1R by immunohistochemistry, we used a highly specific antibody that yields no labeling in the brain of *Glp1r* knock-out mice^14,16,29^. The distribution of immunohistochemically labeled GLP-1R perikarya was identical to the distribution of *Glp1r* transcript-expressing cells. In addition to cell bodies, immunohistochemistry also detected GLP-1R-immunoreactivity in dendrites and axons. As described in the Introduction, previous studies with little or no antibody specificity control experiments reported GLP-1R immunoreactivity primarily in glial cells, and to a lesser degree in neurons^24–28^. These studies showed homogenous^26,28^ or “spotty”^25,27^ intracellular GLP-1R-IR signal in neurons. In contrast, we observed GLP-1R-labeling prominently along the cell membrane, as well as intracellularly, often distinctly in membrane compartments that is in agreement with the membrane receptor nature of GLP-1R. This subcellular distribution, the use of a highly specific antibody, and the identical distribution of the GLP-1R-IR and *Glp1r* mRNA-containing perikarya provide strong evidence that the neuronal GLP-1R expression pattern described here is specific. At the ultrastructural level, we detected GLP-1R in neuronal dendrites and axons in addition to its perikaryal localization, as previously observed in the forebrain^16^. In addition to the neuronal localization of the receptor, we detected GLP-1R-immunoreactivity in a few glial processes at the ultrastructural level, and observed *Glp1r* mRNA in primary cultures of astrocytes. However, without corroborating evidence that *Glp1r* mRNA or GLP-1R is expressed in glial cells in the adult spinal cord, the presence of GLP-1R in glial cells requires further investigation.

In agreement with the fiber distribution pattern of *Gcg* neurons described by Llewellyn-Smith and colleagues^20^, we report that GLP-1 axons provide moderate to dense innervation to most areas where GLP-1R neurons are present, except laminae II–III of the dorsal horn. While the vast majority of GLP-1 axons in the spinal cord evidently originates from lower brainstem neurons^9,20^, PPG-promoter driven transgene expression suggested that GLP-1 may be produced locally by a small population of neurons^20^. We verified that *Gcg* is expressed in scattered neurons in the lumbo-sacral segments, and also identified such neurons in the cervical enlargement (which was not examined in PPG-YFP mice^20^). These neurons, however, express very low *Gcg* mRNA levels compared to *Gcg* in the lower brainstem, and it remains to be determined whether they may serve as a functionally relevant source of GLP-1 in local spinal cord circuits.

### The GLP-1/GLP-1R system in laminae II–III

The mismatch between the high GLP-1R content and the very sparse GLP-1 innervation of laminae II–III raises the question as to how and from what source the ligand of GLP-1R may reach the neurons of this region. GLP-1 may spread into laminae II–III from other, more densely innervated laminae by volume transmission through the neuropil. However, it is also conceivable that circulating GLP-1 can enter these laminae through the blood-brain-barrier as Fu et al.^39^ showed that peripherally injected GLP-1 rapidly enters the brain through endothelial cells. Alternative explanations include that GLP-1R in lamina II–III neurons is a standby receptor for inducible GLP-1 expression under certain conditions (e.g., injury), or binds a yet unknown ligand.

In addition to the GLP-1R-expressing perikarya in laminae II–III, this region also receives very dense GLP-1R-containing innervation. Since many neurons of laminae II–III are local interneurons^40^, it is likely that a proportion of the GLP-1R input originates from local interneurons. Activation of spinal cord GLP-1Rs was shown to suppress pain hypersensitivity^24^, suggesting that GLP-1R signaling in laminae II–III may be important in the regulation of the processing of sensory information.

### Interaction of the GLP-1/GLP-1R and cholinergic systems

To the best of our knowledge, the only physiological context in which the GLP-1 innervation of the spinal cord has been implicated is the regulation of heart rate via SPNs^20,21^. Holt and colleagues^21^ demonstrated that the stimulation of NTS GLP-1 neurons, as well as the direct application of GLP-1 onto the spinal cord increases heart rate. In addition, the positive chronotropic effects of systemically administered GLP-1R agonists, such as exendin-4 or liraglutide, depend on increased sympathetic activity in rodents^21,41,42^. Thus, direct GLP-1R signaling in SPNs might have clinical relevance as it may account for increased heart rate, a common side effect of GLP-1R agonist medications^21^.

We observed the only well-defined and sizable population of GLP-1R-expressing SPNs in the rostral IML, in the C8-T2 segments. The center of this cell group was in T1, comprising about 25% of IML SPNs in this segment. In rodents, SPNs in the C8-T2 segments innervate primarily the superior cervical and middle cervical-stellate ganglion complex^43–49^, from which postganglionic neurons innervate the heart, salivary glands and the eyes, among other targets^47,50–52^. Furthermore, anatomical and physiological studies indicate that a subset of IML SPNs in the T1–T2 segments innervates cardiac postganglionic neurons^53,54^. Further anatomical studies are required to confirm whether GLP-1R^+^ SPNs in the C8-T2 IML innervate cardiac postganglionic neurons.

Interestingly, we found that the IML in C8-T2 does not receive a more robust GLP-1 innervation than in other segments where SPNs do not express GLP-1R, and GLP-1 fibers seldom contacted the cell bodies or proximal dendrites of GLP-1R^+^ SPNs. However, SPNs in the T1–T3 IML have multiple long dendritic branches that extend rostro-caudally, laterally, and also medially towards the central canal where they even form a subependymal plexus^48^. These dendritic branches might be the primary receivers of GLP-1 signaling, as GLP-1 fibers are denser around the central canal, and GLP-1 also appears to be transmitted via the CSF^55^. Of note, the tachycardia reported by Holt and colleagues was elicited by GLP-1 applied to the T7/T8 segments^21^. While this stimulus was rather distant from the C8-T2 segments and could involve GLP-1R^+^ interneurons (see below), it is conceivable that dendrites of GLP-1R^+^ SPNs extending well into the mid-thoracic segments mediated this effect.

A very small group of *Glp1r*^+^ *Chat*^+^ neurons was present in the caudal IML, in the T12-L1 segments. SPNs from these segments project primarily to the mesenteric ganglia^43^, affecting organs in the pelvic viscera, such as the gonads and colon^36^. While no *Glp1r*^+^ *Chat*^+^ neurons were observed in the IML in other segments, a population of non-cholinergic IML neurons express high GLP-1R levels. These are likely local interneurons that affect the activity of SPNs^36,56,57^. Therefore, brainstem GLP-1 neurons may regulate additional SPN populations via these interneurons. We also hypothesize that GLP-1 axons that are apposed to GLP-1R-negative SPNs may regulate these cells directly via synaptic glutamate release^58,59^, and indirectly by GLP-1 release that might modulate the activity of nearby GLP-1R^+^ terminals on the surface of SPNs^60^.

GLP-1R^+^ ChAT^+^ neurons were also observed in the other two autonomic nuclei, the ICN and CAA. While cholinergic neurons in the ICN are considered SPNs, they can be either SPNs or interneurons in the CAA^36^. Further studies are needed to determine whether cholinergic GLP-1R neurons in the CAA include SPNs, and whether they constitute a functionally significant population to mediate the effects of GLP-1-signaling toward end organs.

It is worth noting that since SPNs project outside the blood-spinal cord barrier^61^, systemically injected GLP-1R agonists may easily access GLP-1Rs on the axon terminals of these cells in the sympathetic ganglia. Thus, mild tachycardia caused by GLP-1R agonists^21^ may be induced by the activation of GLP-1Rs on SPN axons innervating cardiac postganglionic neurons. Similarly, GLP-1R activation on the axons from dispersed SPNs in the ICN, and possibly CAA, may account for other, rarer side effects of GLP-1R agonists.

*Glp1r* was only occasionally expressed in other cholinergic cell groups. These data comport well with a recent RNA-Seq dataset obtained from spinal cord cholinergic neurons that detected *Glp1r* transcript in only small fractions of visceral and skeletal motor neurons, and interneurons^62^.

### CSF-contacting neurons as GLP-1-receptive cells

Our study identified CSF-contacting neurons as the cells expressing possibly the highest GLP-1R protein level in the spinal cord. This result is in agreement with RNA-Seq data that found high levels of *Glp1r* transcript in CSF-contacting neurons in the developing mouse spinal cord^22^. CSF-contacting neurons are sensory neurons, able to detect CSF motion as well as changes in its chemical composition via their central dendrite protruding into the CSF^30^. The intense GLP-1R immunolabeling in these dendritic bulbs suggests that CSF-contacting neurons monitor the GLP-1 content of the CSF. Since the primary, if not exclusive, source of GLP-1 in the CSF are brainstem GLP-1 neurons (and not GLP-1 from the circulation^3^), this suggests that CSF-contacting neurons are regulated by the overall activity of brainstem GLP-1 neurons. GLP-1 axons also formed close appositions with many CSF-contacting neurons. Further studies are required to uncover what aspects of physiology GLP-1 regulates via CSF-contacting neurons.

### Summary

Here we characterized the GLP-1/GLP-1R system in the spinal cord of male mice, by providing the expression/distribution pattern for both the receptor and ligand, and identifying specific neuron populations that express GLP-1R. We believe the results will facilitate further physiological studies to better understand how GLP-1R agonists regulate autonomic and somatic functions in the spinal cord.

## Methods

### Animals

Adult male C57Bl/6 J mice were used from the local colony of the Institute of Experimental Medicine, Hungary. The number and details of animals are listed below for each procedure. Animals were housed together (4–5 in a cage) under standard environmental conditions (lights on between 06:00 and 18:00 h, temperature 22 ± 1 °C, mouse chow and water ad libitum). All experiments were carried out in accordance with the Directive 2010/63/EU of the European Union on the protection of animals used for scientific purposes. All experimental protocols were reviewed and approved by the Animal Welfare Committees at the Institute of Experimental Medicine. The study design and all procedures followed the ARRIVE Guidelines.

### Tissue preparation for fluorescent in situ hybridization (FISH)

Nine male C57Bl/6 J mice (six 8 weeks old, two 14 weeks old, one 20 weeks old, 22–35 g) were anesthetized with the mixture of ketamine and xylazine (ip, ketamine 50 mg/kg, xylazine 10 mg/kg body weight) and then decapitated. The spinal cords were ejected from the spinal canal into ice-cold RNase-free PBS, based on the protocol described by Kennedy et al.^63^. In our experience, the pressure ejection of the whole spinal cord could easily damage the middle of the cord (central canal area) in the lower cervical/upper thoracic segments. This could be largely prevented by cutting off the upper spine above the cervical enlargement (above C5), therefore we cut off this part in some cords to preserve tissue integrity. Table 1 includes the starting cervical segment for 8 cords; the ninth cord started with C3. Following ejection, the spinal cord was cut into three segments (cervical-upper thoracic, middle thoracic-lumbar, lumbar-coccygeal) with fine scissors, and transferred into a cryomold, filled with optimal cutting temperature (OCT) medium (Leica Microsystems, Nussloch GmbH, Germany). The OCT-embedded parallel segments were frozen on the surface of ethanol pre-cooled with dry ice, and stored at − 80 °C until sectioning. The brains and adjoining upper cervical cord (C1-2 segments) were also removed from two mice, and quickly frozen on powdered dry ice. Then, 16 μm thick coronal sections were cut using a Leica CM3050 S cryostat (Leica Microsystems), thaw-mounted on Superfrost Plus glass slides (Fisher Scientific Co., Pittsburgh, PA), and air-dried. Sections were collected at 1 mm intervals (every 62nd section) through the entire length of the cord segments and placed on the same slide. Eight representative series of sections were collected in this way from each spinal cord, and stored at − 80 °C until processed for FISH. From the lower brainstems, every 8th 16 μm thick coronal cryosection was collected and stored as described above.

### Single-label FISH for Glp1r or Gcg

Spinal cord cryosections collected at 1 mm interval were hybridized with digoxigenin-labeled riboprobes for *Glp1r* (corresponding to 11-1402 of the mouse *Glp1r* mRNA, Genbank # NM_021332.2)^60^ or *Gcg* (corresponding to 75-944 of the mouse *Gcg* mRNA, NM_008100.4). Lower brainstem sections were hybridized for *Gcg*. The *Gcg* template cDNA fragment was generated with RT-PCR from a murine brainstem sample using standard procedures, cloned into pGemT vector (Promega), and confirmed by sequencing. The FISH procedure followed the protocol previously described for fresh frozen sections^64^, except that lower concentration of RNase A (5 µg/ml) was used in the post-hybridization phase. Following post-hybridization stringency wash steps, sections were treated with 0.5% Triton X-100/0.5% H_2_O_2_ in PBS for 15 min, rinsed in PBS, immersed in maleate buffer (0.1 M maleic acid, 0.15 M NaCl, pH 7.5; 10 min), and in 1% blocking reagent for nucleic acid hybridization (Roche Applied Sciences, Basel, Switzerland). The sections were incubated overnight in peroxidase-conjugated sheep anti-digoxigenin antibody Fab fragments (Roche, Cat# 11207733910; diluted 1:100 in 1% blocking reagent) using CoverWell incubation chambers (Grace Bio-Labs Inc., Bend, OR). The hybridization signal was amplified with the TSA Plus Biotin Kit (Akoya Biosciences, Cat# NEL749A001KT) for 30 min, using the TSA Plus biotin reagent at 1:500 dilution in 0.05 M Tris containing 0.01% H_2_O_2_. The biotin deposits were detected with Alexa Fluor 488-conjugated Streptavidin (ThermoFisher, 1:500).

### Post-FISH immunofluorescence

Sections hybridized for *Gcg* were incubated overnight in a Guinea pig antiserum against NeuN (Millipore, Cat# ABN90; 1:2000 dilution), and then in Cy3-conjugated anti-Guinea pig IgG (Jackson Immunoresearch, 1:200) for 2 h. Sections hybridized for *Glp1r* were incubated overnight in the cocktail of the Guinea pig NeuN antiserum and either a rabbit antiserum against polycystin-L (PKD2L1) (Millipore, Cat# AB9084, 1:500) or a mouse monoclonal antibody against GFAP (Millipore, Cat# MAB360, 1:4000). The primary antibodies were detected with Alexa Fluor 555-conjugated donkey anti-rabbit or anti-mouse IgG (ThermoFisher; 1:500 each), and Cy5-conjugated donkey anti-Guinea pig IgG (Jackson, 1:200). All antibodies were diluted in antibody diluent (2% normal horse serum, 0.2% Photo-flo, 0.2% sodium azide in PBS). Sections were coverslipped with SlowFade Diamond mountant containing DAPI (ThermoFisher). Since the PKD2L1 immunolabeling was very weak following FISH, we repeated the hybridization procedure at lower temperatures (50 °C for overnight hybridization, 60 °C post-hybridization washing steps), which greatly improved the intensity of PKD2L1 immunolabeling, while not reducing the quality of FISH.

### Dual-label FISH for Glp1r and Chat

The spinal cord sections were hybridized with the cocktail of the digoxigenin-labeled antisense *Glp1r* riboprobe, and a 2326 bases long fluorescein-labeled antisense *Chat* riboprobe transcribed from a cDNA template corresponding to the full-length rat *Chat* mRNA (Genbank # XM_032919318.1) (kind gift from Sylvie Berrard, Université de Paris, NeuroDiderot, Inserm, Paris, France). The FISH procedure was identical to the single FISH experiment described above. The sections were first incubated overnight in peroxidase-conjugated sheep anti-digoxigenin antibody Fab fragments, and then the *Glp1r* hybridization signal was amplified using the TSA Plus DIG Kit (Akoya Biosciences, Cat# NEL748E001KT) for 30 min, applying the DIG amplification reagent at 1:500 dilution in 0.05 M Tris (pH 7.6) containing 0.01% H_2_O_2_. Sections were then incubated in a rabbit monoclonal anti-digoxigenin antibody (ThermoFisher, Cat #700772; at 1 μg/ml concentration) for 2 h, in the presence of 2% sodium azide to inactivate peroxidase activity. Sections were then incubated overnight in peroxidase-conjugated sheep anti-fluorescein antibody Fab fragments (Roche, Cat# 11426346910, diluted 1:100 in 1% blocking reagent). The *Chat* hybridization signal was amplified with the TSA Plus Biotin Kit for 30 min as described above. Sections were subsequently incubated in the cocktail of Alexa Fluor 488-conjugated Streptavidin (ThermoFisher, 1:500) and Alexa Fluor 555-conjugated donkey anti-rabbit IgG (ThermoFisher; 1:500) for 2 h, then coverslipped with SlowFade Diamond mountant containing DAPI.

### Tissue preparation for light microscopic immunohistochemistry/immunofluorescence

Male C57Bl/6 J mice (n = 3, 12–13 weeks old, weighing 27–31 g) were anesthetized with a mixture of ketamine and xylazine as described above, and perfused transcardially with 10 ml PBS (pH 7.4), followed by 40 ml 4% paraformaldehyde in 0.1 M PB (pH 7.4). The spinal cord was carefully removed by laminectomy, postfixed for 4 h in 4% PFA and transferred into 30% sucrose overnight for cryoprotection. The spinal cord was then cut into four rostro-caudal pieces, embedded in OCT in a cryomold, and rapidly frozen on ethanol pre-cooled with dry ice. The parallel pieces were cut on a cryostat into 1-in-4 series of 25 µm thick coronal sections and stored in anti-freeze solution (30% ethylene glycol, 25% glycerol, 0.05 M PB) at − 20 °C until used.

### Light-microscopic chromogenic immunohistochemistry

*GLP-1R immunohistochemistry*. Every fourth section from 2 mm long segments of the cervical, thoracic, lumbar and sacral regions were treated with a mixture of 0.5% Triton X-100 and 0.5% H_2_O_2_ in PBS for 15 min. After rinses in PBS, the sections were incubated in antibody diluent (defined above) for 20 min to reduce non-specific antibody binding. The sections were incubated in a rabbitized mouse monoclonal antibody against GLP-1R (clone 7F38, Novo Nordisk A/S, Denmark; 0.015 µg/ml)^16^, overnight at room temperature. After rinses in PBS, the sections were incubated in biotinylated donkey anti-rabbit IgG for 2 h, followed by incubation in avidin–biotin complex (ABC Elite, PK-6100, 1:1000, Vector Laboratories Ltd, UK) for 1 h. The immunoreaction was detected with Ni-DAB developer (0.05% diamino-benzidine, 0.15% Ni-ammonium-sulfate and 0.005% H_2_O_2_ in 0.05 M Tris buffer, pH 7.6). The sections were mounted onto glass slides, dehydrated in ascending series of ethanol and xylenes, and coverslipped with DPX mounting medium (Merck KGaA, Darmstadt, Germany).

*GLP-1 immunohistochemistry*. Since we found that the GLP-1 antibody worked superior (with less background) on the acrolein/PFA-perfused tissue prepared and cut for immuno-electron microscopy (see below), we used those sections for chromogenic GLP-1 immunohistochemistry. We first treated the sections with 1% sodium borohydride in distilled water for 30 min, and then applied the same treatments described above. Sections were incubated overnight in a mouse monoclonal antibody against GLP-1 (clone 62-2F6, Novo Nordisk A/S; 2 µg/ml)^60^, then in biotinylated donkey anti-mouse IgG (Jackson, 1:500) for 2 h. The rest of the procedure was the same as described above for GLP-1R immunohistochemistry.

### Immunofluorescence

Sections were pretreated as described above, and dual or triple immunofluorescence was performed using overnight incubation for the cocktail of primary antibodies, and 2–3 h incubation for the secondary antibodies. Primary antibodies for the first staining were: rabbitized GLP-1R antibody (0.12 µg/ml), Guinea pig NeuN antiserum (1:2000), and mouse GFAP antibody (1:4000). The secondary antibodies were Alexa 488-conjugated donkey anti-mouse, Alexa 555-conjugated donkey anti-rabbit, and Cy5-conjugated donkey anti-Guinea pig IgGs. For the second staining, the primary antibodies were: rabbitized GLP-1R antibody (0.12 µg/ml), mouse GLP-1 antibody (0.5 µg/ml), and a goat antiserum against ChAT (Millipore, Cat# AB144P; 1:2000). The secondary antibodies were Alexa 488-conjugated donkey anti-sheep, Alexa 555-conjugated donkey anti-rabbit and Alexa 647-conjugated donkey anti-mouse IgGs. In the third staining, the primary antibodies were rabbitized GLP-1R antibody (0.12 µg/ml) and a goat polyclonal antiserum for Iba1 (ThermoFisher, Cat# PA5-18039; 1:250), detected with Alexa 555-conjugated donkey anti-rabbit and Alexa 488-conjugated donkey anti-sheep IgGs. Sections were coverslipped with SlowFade Diamond mountant containing DAPI.

### Imaging

Chromogenic immunohistochemistry images and low-magnification fluorescent images were taken with a Zeiss Axio Imager M2 epifluorescent microscope equipped with AxioCam MRc5 (Carl Zeiss, Germany) and AxioVision Se64 Rel.4.9.1 software. Higher magnification fluorescent images were taken with a Zeiss LSM 780 confocal microscope (Zeiss Company, Germany). We used line-by-line sequential scanning with laser excitation lines 405 for DAPI, 488 nm for Alexa Fluor 488, 561 nm for Alexa Fluor 555 or Cy3, and 633 nm for Alexa 647 or Cy5, with MBS 488/561/633 and MBS -405 beamsplitters. Detection wavelengths were set for 410–497 nm for DAPI, 499–552 nm for Alexa 488, 570–624 nm for Alexa 555 or Cy3, and 638–755 nm for Alexa 647 or Cy5. Plan-Apochromat 20 × and 63 × lenses were used, with pinhole sizes set to obtain 1.5–1.9 and 0.7–0.8 µm thin optical slices, respectively. Confocal Z-stack images were recorded and analyzed with Zen 2012 software (Zeiss Company, Germany). Adobe Photoshop was used to create (Adobe System Inc., USA) composite images and to modify brightness and contrast for illustrations.

### Cell counts

*Glp1r*^+^ *Chat*^+^ neurons and *Gcg* neurons (FISH studies) were counted bilaterally, on serial 16 µm thick cryo-sections collected at 1 mm interval. As this is approximately the length of a spinal cord segment, essentially each segment was represented by a single section. This, and that the linear order of sections was kept, allowed the precise identification of spinal cord segments in the FISH studies. Cells were counted manually with the epifluorescent microscope; for a few ambiguous cells, co-localization of the *Glp1r* and *Chat* hybridization signals were confirmed with confocal microscopy. *Gcg* neurons were counted in the same cords shown in Table 1, with the exception that instead of cord # 7371, another cord was examined that started with the C3 segment and ended in the coccygeal part.

GLP-1R^+^ ChAT^+^ and single ChAT^+^ neurons (immunofluorescence) were counted in the T1 IML from four 25 µm-thick sections, on confocal Z-stack images obtained with the 63 × objective. The T1 segment was identified based on the shape of the gray matter and distribution pattern of ChAT neurons.

### Immuno-electron microscopy

Under ketamine/xylazine anesthesia as described above, mice (n = 3; 6, 10 and 14 week old) were perfused transcardially with 10 ml PBS followed by 50 ml fixative containing 3% PFA and 1% acrolein (Merck, Germany) in 0.1 M PB. The spinal cord was removed after opening the spinal canal by laminectomy and postfixed overnight in 4% PFA in PBS. The spinal cord was cut into shorter pieces, embedded in agar, and 25 µm thick coronal sections were cut on a Leica VT 1000S vibratome (Leica Microsystems, Austria). Sections were stored in anti-freeze solution at − 20 °C until used. Sections were washed with PBS and treated with 1% sodium borohydride in 0.1 M PB (pH 7.4) for 30 min and then with 0.5% H_2_O_2_ in PBS for 15 min. Sections were cryoprotected in 15% sucrose solution in PBS for 30 min, followed by 30% sucrose solution in PBS overnight at 4 °C. Then sections were quickly frozen over liquid nitrogen and thawed. The freeze-thaw cycle was repeated three times, to improve antibody penetration. The sections were then incubated in antibody diluent for 20 min to prevent non-specific antibody binding. The pretreated sections were incubated in the rabbitized GLP-1R antibody (0.015 µg/ml) for 4 days at 4 °C. After rinses in PBS, the sections were incubated overnight in biotinylated donkey anti-rabbit IgG (Jackson, 1:500), and then in ABC (1:1000) for 1 h. GLP-1R immunoreactivity was detected with Ni-DAB developer, and in some sections the reaction product was silver intensified using the Gallyas method^65^. The immunolabeled sections were incubated in 1% osmium-tetroxide (Electron Microscopy Sciences, USA) for 1 h at room temperature, then treated with 2% uranyl acetate in 70% ethanol for 30 min; followed by dehydration in an ascending series of ethanol and acetonitrile (Merck, Germany). Then, sections were flat embedded in Durcupan ACM epoxy resin (Merck, Germany) on liquid release agent-coated slides (Electron Microscopy Sciences, USA), and polymerized at 56 °C for 2 days. Ultrathin, 60–70 nm thick sections were cut with Leica UCT ultramicrotome (Leica Microsystems, Germany). The ultrathin sections were mounted onto Formvar-coated, single slot grids, treated with lead citrate, and examined with a JEOL-100 C transmission electron microscope.

### Glp1r RT-PCR in primary cultures of mouse spinal cord astrocytes

Primary cultures of astrocytes were established from the spinal cord of 3-day-old C57Bl/6 J male mice following standard procedures^66^. In short, the samples were put in ice-cold PBS and centrifuged for 20 s at 100 g at room temperature followed by washing three times with 37 °C PBS and by digestion in trypsin and DNase at 37 °C for 10 min. Cells were suspended in MEM, centrifuged for 10 min at 100 g then cultured in medium (MEM [Gibco], 10% FBS [Gibco], 4 mM L-glutamin [Sigma] + 2.5 µg/ml amphotericin B [Gibco], 0.04 mg/ml Gentamicin [Gentamicin Sandoz 80 mg/ml]) for 14 days at 37 °C, 5% CO_2_. RNA was isolated with the Picopure RNA isolation Kit (ThermoFisher Scientific) followed by reverse transcription by Applied Biosystem High Capacity cDNA kit. Taq PCR was performed to amplify the *Glp1r* cDNA using the following intron-spanning primers, as described by Xu et al.^67^: sense, GGGTCTCTGGCTACATAAGGACAAC; antisense, AGCCTTCAGTTTGGAGACCACTAT generating a 690 nt amplicon (annealing 55 °C, 36 cycles). A mouse arcuate nucleus sample and water were used as templates for positive and negative controls, respectively.

### Flow cytometric analysis of spinal cord microglia

For flow cytometric analysis, cells were isolated from the spinal cord of adult CX3CR1^GFP/+^ (RRID:IMSR_JAX:005582) mice (n = 5). Following dissection, the tissues were minced with a scalpel blade and Dounce homogenized 2 × 15 − 20 times, passed through a pre-wet 70-μm filter and centrifuged at 300 g for 8 min, at 4 °C. The cell pellet was resuspended in 40% Percoll (#17-0891-01, GE Healthcare) diluted in calcium and magnesium free HBSS and spun for 1 h at 500 g and 4 °C in a swinging bucket centrifuge for myelin removal. Microglia were then washed with HBSS by centrifugation for 5 min at 500 g at 4 °C and resuspended in FACS buffer (0.1% BSA in PBS; sterile filtered). For immunolabeling, cells were incubated with anti-mouse CD16/32 (eBioscience Ref 16-0161-85, 1:100) for 15 min to block Fc receptors. The rabbitized GLP-1R antibody (see above) was then applied for 30 min at 1.8 or 0.36 µg/ml dilution together with a microglia specific marker (anti-mouse Tmem119-PE-Cy7; Invitrogen Ref 25-6119-80, 1:200). Finally, donkey anti-rabbit Alexa 594 (711-586-152, Jackson) was added to the cell suspensions at 1:100 dilution, for 30 min. All staining steps were performed on ice. Samples were acquired on a BD FACSVerse flow cytometer and data analyzed using FACSuite software (BD, Becton, Dickinson and Company).

### Supplementary Information

Supplementary Figures.

## Data Availability

The data generated during the current study are available from the corresponding authors on reasonable request.
