# Supplementary material for: Topography of the GLP-1/GLP-1 receptor system in the spinal cord of male mice
Source: Sci Rep. 2024 Jun 22;14:14403. doi: 10.1038/s41598-024-65442-1 (PMC11193760; doi:10.1038/s41598-024-65442-1)
Supplement: Supplementary file 1 — Supplementary Figures. [file 41598_2024_65442_MOESM1_ESM.pdf]

Topography of the GLP-1 / GLP-1 receptor system in the spinal cord of male mice

Yvette Ruska, Andrea Csibi, Beáta Dorogházi, Anett Szilvásy-Szabó, Petra Mohácsik, Zsuzsanna Környei, Ádám Dénes, Andrea Kádár, Zita Puskár, Erik Hrabovszky, Balázs Gereben, Gábor Wittmann, Csaba Fekete

Supplementary Figures

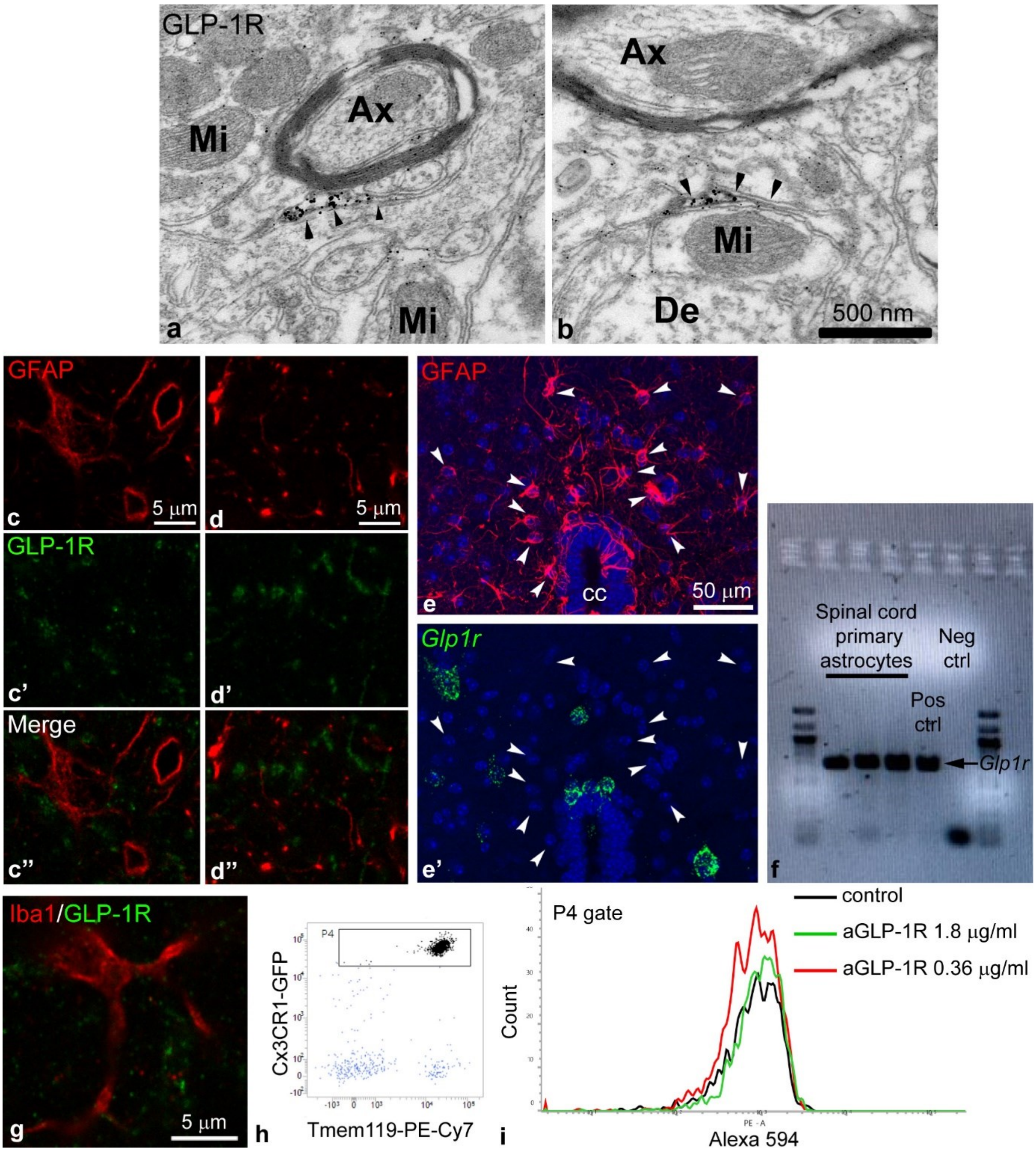

Supplementary Figure S1. Mismatching data about the GLP-1R expression in spinal cord glial cells. a-b) Immuno-electron microscopy for GLP-1R shows immunoreaction (silver-intensified Ni-DAB, arrowheads) inside thin processes likely of glial origin. **c-d'')** Dual immunofluorescence for GLP-1R (green) and the astrocyte marker GFAP (red) shows the lack of GLP-1R in the cell body (**c-c''**) and processes (**c-c''**, **d-d''**) of astrocytes. **e-e'**) *Glp1r* FISH (green) combined with GFAP immunofluorescence (red) shows the lack of *Glp1r* transcript in astrocytes (arrowheads). DAPI is shown in blue. f) RT-PCR shows *Glp1r* mRNA expression in primary astrocyte cultures isolated from the spinal cords of P3 mice. The positive control is an arcuate nucleus sample from an adult mouse, the negative control does not contain cDNA. **g**) Dual immunofluorescence for GLP-1R (green) and the microglia marker Iba1 (red) shows no GLP-1R expression in a microglia. **h**) Flow cytometric dot plot identifies spinal cord

microglia of adult Cx3CR1$^{GFP/+}$ mice labeled with Tmem119-PE-Cy7 (P4 gate). **i**) Histogram showing no cell surface presentation of GLP-1R on microglia. The primary antibody was applied at 1.8 or 0.36 μg/ml dilutions. Control (black) cells were incubated with secondary Alexa 594 antibody without the primary antibody. Abbreviations: Ax, axon; cc, central canal; De, dendrite; Mi, mitochondrium.

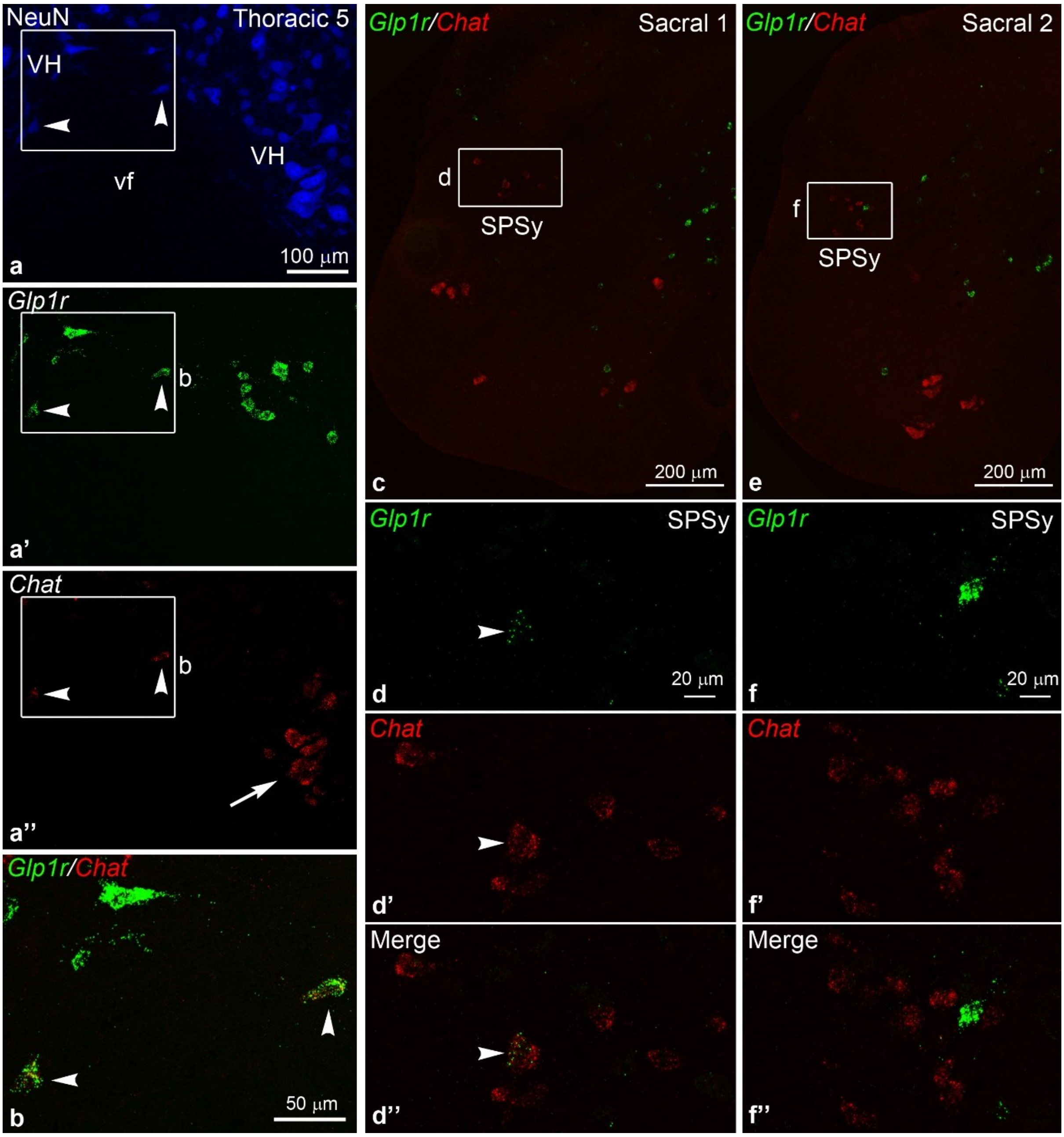

Supplementary Figure S2. ***Glp1r*** **is expressed in rare cholinergic neurons in lamina VIII and the sacral parasympathetic nucleus. a-a’’)** Dual FISH for *Glp1r* (green) and the cholinergic marker *Chat* (red) in the T5 segment. NeuN immunofluorescence in **a** (blue) helps identifying the ventral horn and ventral funiculus. Arrowheads indicate two *Glp1r*$^{+}$ *Chat*$^{+}$ neurons in lamina VIII at the medial border of the ventral horn. The arrow in **a’’** indicate large *Chat*$^{+}$ somatic motor neurons in lamina IX that do not express *Glp1r*. **b)** Magnified and merged view of the boxed area from **a’-a’’** shows the two *Glp1r*$^{+}$ *Chat*$^{+}$ neurons (arrowheads). **c-f’’)** *Glp1r*$^{+}$ *Chat*$^{+}$ neurons are extremely rare in the sacral parasympathetic nucleus. **c** and **e** show low magnification fluorescent images of the S1 and S2 segments. The parasympathetic nucleus (boxed areas) is shown in higher magnification confocal images (Z projections, 7-8 µm total thickness) in **d-d’’** and **f-f’’**. The arrowhead in **d-d’’** points to a *Glp1r*$^{+}$ *Chat*$^{+}$ neuron; no other *Chat* neurons express *Glp1r*. Abbreviations: SPSy, sacral parasympathetic nucleus; vf, ventral funiculus; VH, ventral horn.

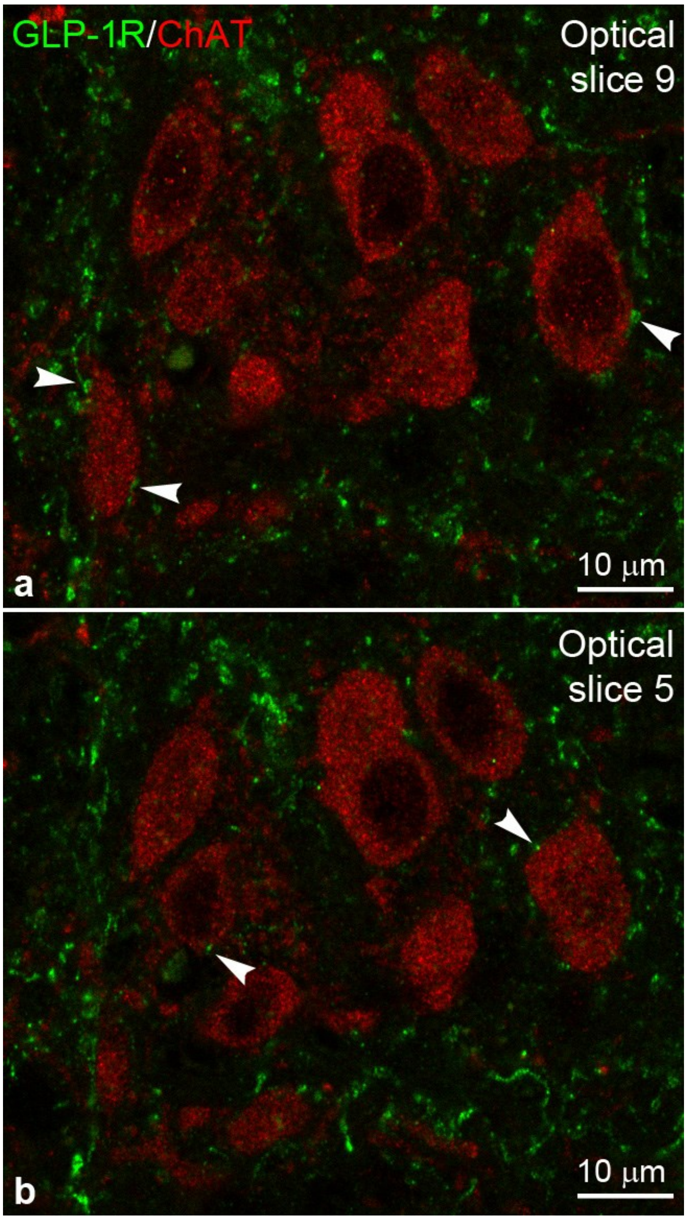

Supplementary Figure S3. ChAT neurons in the IML receive contacts from GLP-1R axons. a-b) Single optical sections (0.8 µm thick, 0.42 µm Z-step) show GLP-1R axon varicosities/segments of different size (arrowheads) in close apposition to ChAT neurons in the T5-6 IML.

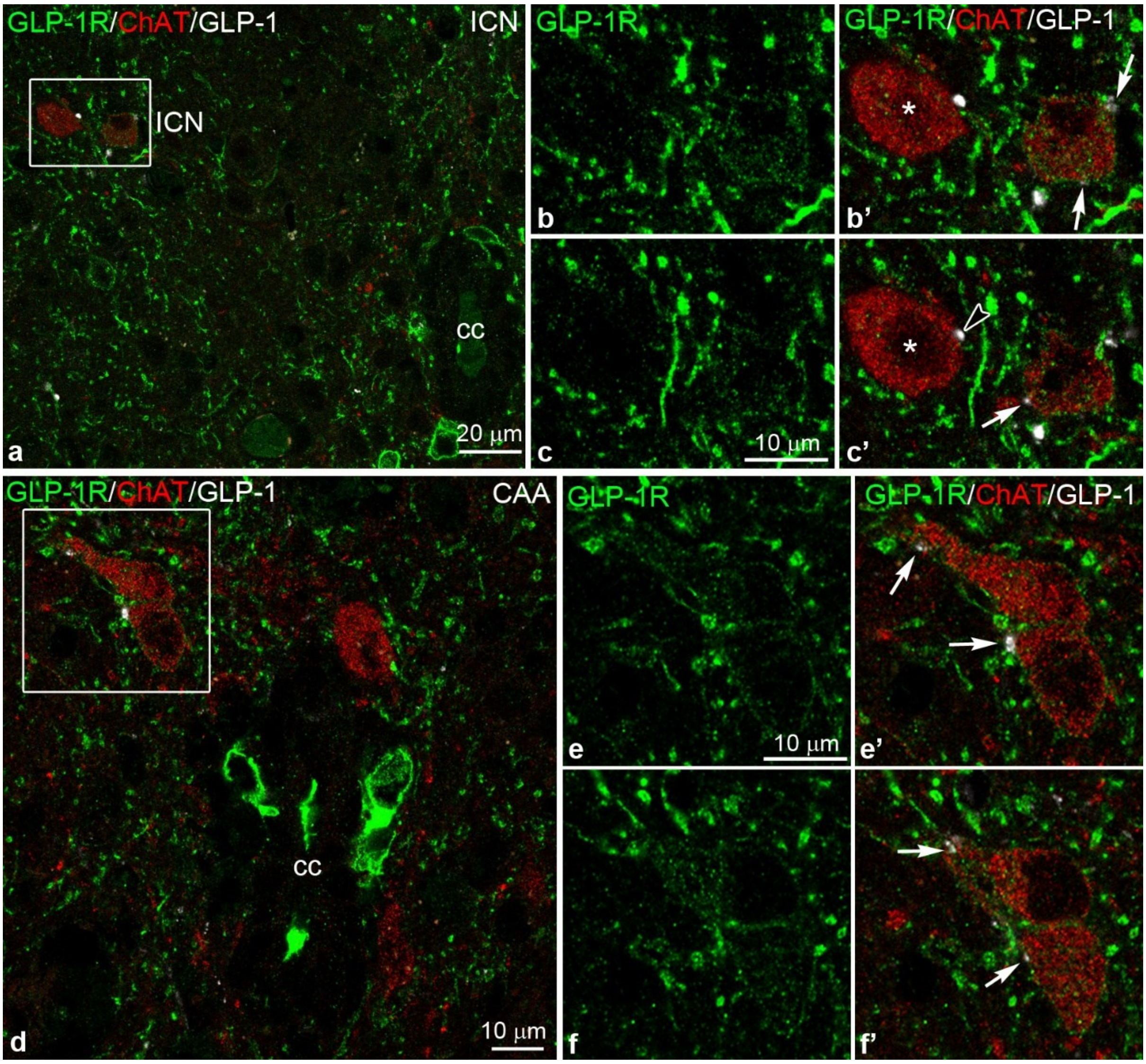

Supplementary Figure S4. GLP-1 axons innervate most GLP-1R$^+$ ChAT neurons in the ICN and CAA. a-c') Two ICN ChAT neurons in the boxed area in **a** are shown in higher magnification in two different optical sections (**b-b'** and **c-c'**). The single optical sections (0.8 µm thick) show that the GLP-1R$^+$ ChAT neuron (on the right) receives close appositions from multiple GLP-1 boutons (arrows). The GLP-1R-negative ChAT neuron (on the left, indicated by *) also receives a close contact from a GLP-1 axon varicosity (open arrowhead). **d-f'**) Two GLP-1R$^+$ ChAT neurons in the CAA in the boxed area in **a** are shown in higher magnification, in two different optical sections (**e-e'** and **f-f'**). GLP-1 varicosities are closely apposed to the proximal dendrite of the upper cell, and to the cell body of the lower cell (arrows). The photos were taken from the T5 (ICN) and T7 segments (CAA). Abbreviations: CAA, central autonomic area; cc, central canal; ICN, intercalated nucleus.

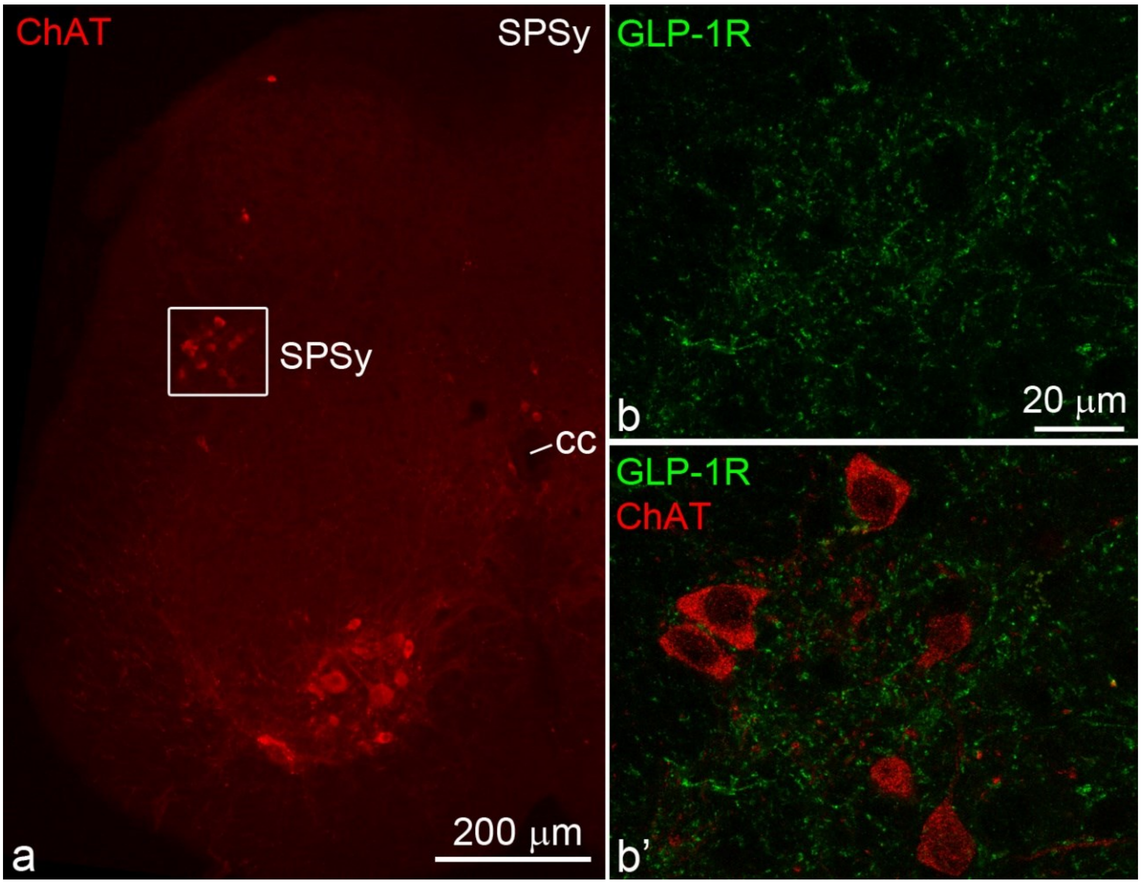

Supplementary Figure S5. ChAT neurons in the sacral parasympathetic nucleus do not express GLP-1R. a) ChAT neurons (red) in the sacral parasympathetic nucleus**. b-b'**) A single optical section (0.8 μm thick) of the boxed area in **a** shows that ChAT neurons do not have GLP-1R (green) on their membrane. Abbreviations: SPSy, sacral parasympathetic nucleus.

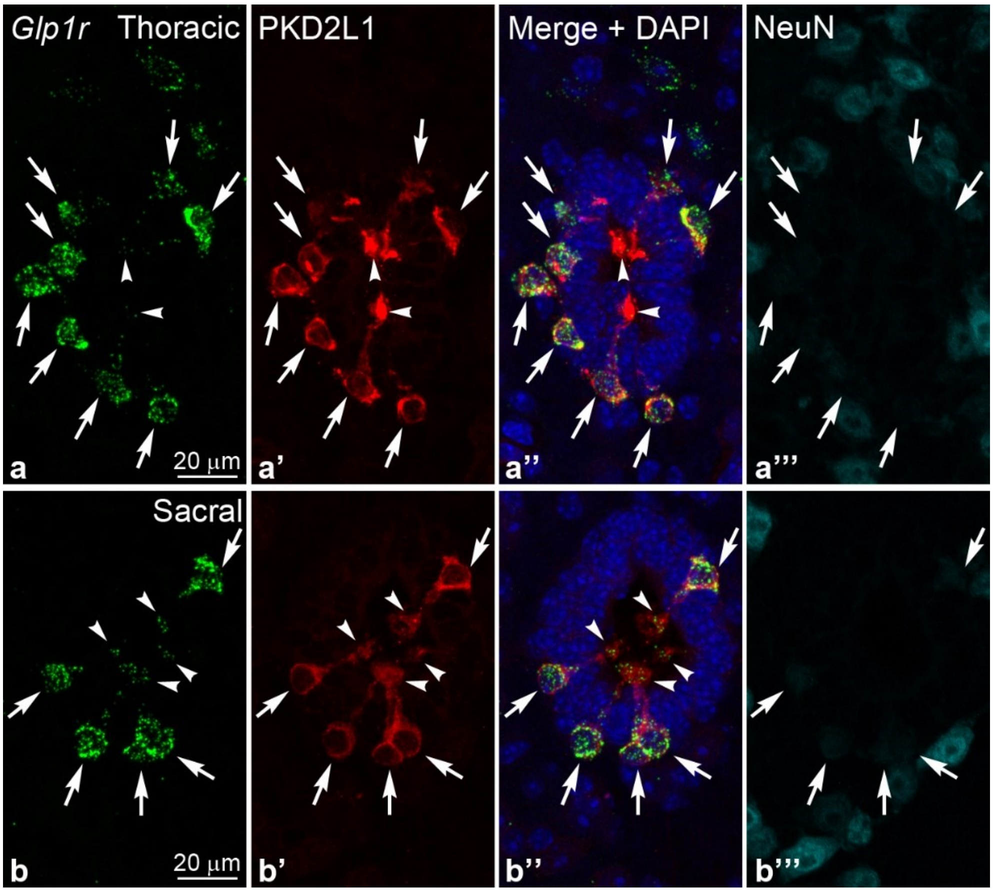

Supplementary Figure S6. CSF-contacting neurons lack or express NeuN at very low levels. a-a’’’) At the thoracic level, CSF-contacting neurons (PKD2L1-positive, red, immunofluorescence) around the central canal express *Glp1r* (green, FISH), but are negative for NeuN (cyan, immunofluorescence) (arrows). **b-b’’’**) At the sacral level, CSF-contacting neurons are lightly positive for NeuN (arrows). Arrowheads point to *Glp1r* mRNA in the central dendritic bulbs. DAPI in blue shows the central canal.
